# Immunoglobulin E-Mediated Autoimmunity

**DOI:** 10.3389/fimmu.2018.00689

**Published:** 2018-04-09

**Authors:** Marcus Maurer, Sabine Altrichter, Oliver Schmetzer, Jörg Scheffel, Martin K. Church, Martin Metz

**Affiliations:** Department of Dermatology and Allergy, Charité – Universitätsmedizin Berlin, Berlin, Germany

**Keywords:** immunoglobulin E-mediated autoimmunity, autoallergy, urticaria, bullous pemphigoid, immunoglobulin E-mechanisms

## Abstract

The study of autoimmunity mediated by immunoglobulin E (IgE) autoantibodies, which may be termed autoallergy, is in its infancy. It is now recognized that systemic lupus erythematosus, bullous pemphigoid (BP), and chronic urticaria, both spontaneous and inducible, are most likely to be mediated, at least in part, by IgE autoantibodies. The situation in other conditions, such as autoimmune uveitis, rheumatoid arthritis, hyperthyroid Graves’ disease, autoimmune pancreatitis, and even asthma, is far less clear but evidence for autoallergy is accumulating. To be certain of an autoallergic mechanism, it is necessary to identify both IgE autoantibodies and their targets as has been done with the transmembrane protein BP180 and the intracellular protein BP230 in BP and IL-24 in chronic spontaneous urticaria. Also, IgE-targeted therapies, such as anti-IgE, must have been shown to be of benefit to patients as has been done with both of these conditions. This comprehensive review of the literature on IgE-mediated autoallergy focuses on three related questions. What do we know about the prevalence of IgE autoantibodies and their targets in different diseases? What do we know about the relevance of IgE autoantibodies in different diseases? What do we know about the cellular and molecular effects of IgE autoantibodies? In addition to providing answers to these questions, based on a broad review of the literature, we outline the current gaps of knowledge in our understanding of IgE autoantibodies and describe approaches to address them.

## Introduction

Friend or Foe? This is the major question that the immune system must address in every individual from the time of conception, through the development of the fetus and the child, right into adulthood. But sometimes it gets this wrong and raises an inappropriate immunological response against self. Although there are several types of autoimmunity this review will address solely immunoglobulin E (IgE)-mediated autoimmunity. Because its mechanisms have much in common with classical allergy involving exogenous allergens, such as grass pollen or house dust mites, IgE-mediated autoimmunity is generally termed “autoallergy” and the target molecules of the response are called “autoallergens.”

Although the presence of an allergic antibody has been postulated since the innovative serum transfer experiment of Prausnitz and Kustner in 1921 (1), it was not until 1967 that this antibody was characterized as IgE (2–4). IgE, which is the least abundant immunoglobulin isotype and found only in mammals, signals through 2 types of Fcε receptor, the high-affinity receptor, FcεRI, which is found primarily on mast cells and basophils, and the low-affinity receptor, FcεRII or CD23, a C-type lectin, which is found on mature B cells, activated macrophages, eosinophils, follicular dendritic cells, and platelets. The primary role of IgE is held to be host defense, particularly initiating the expulsion of parasitic worms (helminths) and environmental substances, such as toxins, venoms, irritants, and xenobiotics.

Although IgE has a well-documented role in classical allergy to exogenous allergens, this review will address only IgE-mediated autoimmunity. It will focus on three related questions: what do we know about the prevalence of IgE autoantibodies and their targets in different diseases? What do we know about the relevance of IgE autoantibodies in different diseases? What do we know about the cellular and molecular effects of IgE autoantibodies? In addition to providing answers to these three questions, based on a comprehensive review of the literature, we outline the current gaps of knowledge in our understanding of IgE autoantibodies and describe approaches to address them.

## What do we know about the Prevalence of IgE Autoantibodies and their Targets in Different Conditions?

Within a decade of its discovery, reports began to incriminate IgE as a possible contributor to the pathogenesis of several chronic inflammatory disorders, such as rheumatoid arthritis (RA) (5–7), bullous pemphigoid (BP) (8, 9), atopic dermatitis (AD) (10), and systemic lupus erythematosus (SLE) (11, 12). More recently, IgE-mediated autoallergy has been suggested for other disorders including chronic spontaneous urticaria (CSU) (13, 14) and chronic inducible urticaria (CindU) (15). The conditions in which IgE autoantibodies have been detected, which are summarized in Table 1, will now be considered in more detail.

**Table 1:** Percentage of patients in which immunoglobulin E (IgE) autoantibodies have been detected in various diseases.

Disease	IgE against	Prevalence	Reference
Allergic rhinoconjunctivitis	Profilin		(16)
Asthma	n.a.	n.a.	(17, 18)
Atopic dermatitis	>140 IgE-binding self-antigens	23–91%	(19, 20)
Autoimmune pancreatitis	n.a.	n.a.	(21)
Bullous pemphigoid	BP 180 or 230	22–100%	(22)
Chronic spontaneous urticaria	Thyroidperoxidase (TPO), double stranded DNA (dsDNA), IL-24	0–80%	(23)
Graves disease	TPO, muscle autoantigens	67%	(24, 25)
Multiple sclerosis (MS)	Small myelin protein-derived peptides	n.a.	(26)
Pemphigus	Desmoglein1, Desmoglein 3, Lamininin-332 und LJM11	11–81%	(22)
Rheumatoid arthritis	ANA, anti-citrullinated protein?	60%	(27)
Systemic lupus erythematodes and lupus nephritis	dsDNA, Sm, SS-A/Ro, SS-B/La, APEX, MPG, CLIP4, ANA, RNP, nucleosome, specific IgE, acidic ribosomal P_2_ protein	3.6–82%	(28)
Uveitis	Retinal S antigen	69%	(29)

*n.a., not assessed*.

### Atopic Dermatitis

Decades before the discovery of IgE, it was reported that human dander extract can elicit immediate-type skin reactions in patients with severe atopy and that this skin sensitivity could be passively transferred with serum (30–35). Many years later, the analysis of the presence of IgE autoantibodies in sera from patients with various manifestations of atopy and other autoimmune disorders indicated that IgE reactivity against a variety of autoantigens occurred most frequently in AD patients. IgE autoreactitvy has first been found and described in AD (36), where IgE autoantibodies are very frequent (23–91%) (19) and especially present in severely affected patients (37–39). IgE autoantibodies had been identified directed against Keratinocytes (40) and a broad variety of autoantigens (41), including, e.g., human protein manganese superoxide dismutase (38), thioredoxin (42), DFS70/LEDGF (43), and Hom s 1–5 (44, 45).

Interestingly, this phenomenon has already been seen in very young infants of less than 1 year where 15% of the children with atopic eczema mounted IgE autoreactivity and raised in the age group of 2–13 years with moderate to severe AE and total IgE serum levels higher 1,000 kU/L up to 80% (46).

### Systemic Lupus Erythematosus

In SLE, several IgE autoantigens have been described (28, 47), and their occurrence is frequent. In a study of 92 SLE patients (48), 29 (32%) had antinuclear-IgE antibodies. No such antibodies were found in a parallel healthy control group. Sub-analysis of the IgE autoantibodies in the 29 positive patients showed reactions with nucleosomes (79.3%), double stranded DNA (dsDNA) (48.3%), SS-A/Ro (48.3%), SS-B/La (18.7%), Sm (48.3%), and RNP (62.1%). In a multicentre study of 196 SLE patients (49), more than 50% showed autoreactive IgE to one or more of the four common SLE nuclear autoantigens, dsDNA, SS-A/Ro, SS-B/La, and Sm. This figure rose to around 74% in patients with active disease where autoreactive IgE to dsDNA was most prevalent with an incidence of 63%. From these findings, Dema and colleagues (49) suggest that the presence of autoreactive IgE, and in particular dsDNA-specific IgE, in SLE, may be a reasonable clinical indicator of increased disease activity. This conclusion is supported by a study that showed that disease flares rates were higher in SLE patients with demonstrable circulating IgE-anti-dsDNA antibodies than in those without (50).

Because approximately half of SLE patients do not have demonstrable IgE antibodies to the common nuclear autoantigens, the prevalence of IgE autoantibodies to other autoallergens has been explored. One study showed high levels of IgE autoantibodies to APEX nuclease 1, *N*-methylpurine-DNA glycosylase and CAP-Gly domain-containing linker protein family member 4 in some SLE patients but not healthy controls. These autoantigens were unique in that they seemed specific targets of IgE autoantibodies but not autoantibodies of the IgG class (49). In another study, 31% of 90 SLE patients displayed IgE antibodies against human P 2 proteins, an antigen that has also been described to be cross-reactive with other members of the ribosomal P 2 protein family, which are minor allergens in fungal allergy (51).

Finally and importantly, IgE to Ro/SSA, a ribonuclearprotein autoantigen has been suggested to be relevant for fetal loss by mothers with SLE (52). This is of special interest as IgG-anti-Ro/SSA antibodies are known to be associated with neonatal lupus (congenital heart block, neonatal transient skin rash, hematological and hepatic abnormalities). However, they are suggested not to negatively affect other gestational outcomes, and the general outcome of these pregnancies is good when managed by experienced multidisciplinary teams (53).

### Bullous Pemphigoid

Bullous pemphigoid is an autoimmune blistering disease that mainly affects elderly patients. It is the most common pemphigoid disorder accounting for around 80% of all pemphigoid cases (28). IgG, IgA, and IgE autoantibodies are directed against two hemidesmosomal proteins, the transmembrane protein BP antigen 2 (BP180, type XVII collagen) and the intracellular BP antigen 1 (BP230) (22, 54, 55). Binding of autoantibodies leads to a complex inflammatory response involving complement activation, the infiltration of inflammatory cells, and the subsequent release of reactive oxygen species and distinct proteases that finally mediate subepidermal splitting (56).

Immunoglobulin E autoantibodies against BP180 target mainly the 16th non-collagenous domain (BP180 NC16A) (54, 57) although BP180 epitopes outside the NC16A domain have also been described to be recognized by IgE (58). That IgE-anti-BP180 is functional was demonstrated by the ability of serum from BP patients to sensitize mast cells and basophils for BP180-induced histamine release (59). The percentage of BP patients with IgE-anti-BP180 autoantibodies, shown in Table 2, varied substantially in the different reports. The reason in this variation is likely to be due to the different test systems used, i.e., ELISA or immunoblotting, and the use of diluted or undiluted patient sera.

**Table 2:** Percentage of patients with bullous pemphigoid showing immunoglobulin E (IgE) reactivity to BP180.

Number of patients	Percentage with IgE-anti-BP180	Reference
37	22	(60)
67	30	(61)
117	40	(56)
44	41	(62)
18	55	(63)
31	61	(64)
56	71	(65)
43	77	(58)
18	83	(66)
10	100	(54)

Several studies were able to detect IgE autoantibodies against the intracellular BP230 autoantigen. Engineer et al. reported IgE-anti-BP230 without detection of BP180-specific IgE autoantibodies in 100% of six BP patients (67). In contrast, other studies revealed IgE-anti-BP230 and IgE-anti-BP180 autoantibodies in 67% of 67 sera (61) and 50% of 32 sera (68). IgE-anti-BP230 reactivity was associated with local eosinophil accumulation (61), with disease activity (69) and correlated with total IgE serum levels (68, 70).

### Other Autoimmune Blistering Disorders

Pemphigus vulgaris (PV) is another severe autoimmune bullous skin disease and is primarily associated with IgG against desmoglein 3 (dsg3), a desmosomal adhesion protein. However, raised IgE in these patients has also been reported (71). Intercellular IgE deposits were detected in the epidermis of 37 patients with acute onset of the disease, and IgE-anti-dsg3 was detected more frequently in the sera of patients with acute onset, compared to chronic-active PV (72). Spaeth and colleagues reported dsg3-reactive IgE in the serum of 13% of 15 patients with acute onset, 11% of 18 patients with chronic-active, and none of 8 patients with remittent PV patients (73).

In mucus membrane pemphigoid, one of four patients showed IgE reactivity to the gamma2 subunit of laminin-332 (74). In endemic pemphigus foliaceus, IgE-anti-desmoglein 1 was found in 81% of 143 patients with fogo selvagem (75), and serum levels of IgE against LJM11, a major immunogenic component of sand fly salivary gland antigens, were significantly higher compared to controls (76).

In summary, reports in pemphigus are limited, but IgE reactivity seems to be more prevalent in the endemic form of pemphigus foliaceus than in PV.

### Chronic Urticaria

An important role of IgE and FcεRI reactions was postulated in both CSU and CindU for several decades (77) before it was finally confirmed by the successful treatment of the disease with anti-IgE (omalizumab) (14, 15, 78). Although specific IgE against classical common allergens, i.e., aeroallergens and food allergens (79–82), or to less common allergens such as fungi (83, 84) can be detected in some patients with CSU, they are not regarded as pathophysiologically relevant for the development of the signs and symptoms of CSU (81, 85–87).

Perhaps the first evidence of autoreacitivitiy in CSU came with the clinical observation that CSU patients have high rates of thyroid diseases (88). Detailed analysis of CSU patients with thyroid pathology led to the detection of IgE anti-thyroidperoxidase (TPO) autoantibodies. Almost 20 years ago, Bar-Sela and coworkers reported the detection of IgE-anti-TPO in a female CSU patient who also had Hashimoto’s thyroiditis (89). More recently, elevated levels of IgE-anti-TPO were reported in 61% of 478 CSU patients (13). However, other studies found only 13–17% of 23 patients (90) and 10% of 20 patients (91) to have IgE-anti-TPO, while a further study failed to find IgE-anti-TPO in 23 CU patients (92). The most likely explanation for these differences is the use of different assay systems, for example direct ELISA vs capture ELISA, and the interference of IgG autoantibodies against TPO (93). Functional relevance was suggested as flow cytometry analysis showed CD203c induction in a dose-dependent manner in basophils sensitized with serial additions of TPO in CU patients having high specific IgE to TPO (94).

Beside thyroid diseases, the prevalence of individual autoimmune diseases in chronic urticaria is increased in general (≥1% in most studies vs ≤1% in the general population). In a review, the rates of comorbidity were ≥1% for insulin-dependent diabetes mellitus, RA, psoriasis, and celiac disease, ≥2% for Graves’ disease, ≥3% for vitiligo, and ≥5% for pernicious anemia and Hashimoto’s thyroiditis. Interestingly, >15% of CSU patients have a positive family history for autoimmune disease (95).

A recent review investigated also the correlation of chronic urticaria with other autoimmune diseases such as SLE. The prevalence of CSU and CSU-like rash in SLE was investigated from 42 independent studies. Comorbidity in adult patients reportedly ranged from 0 to 22% and 0.4 to 28%, respectively (urticarial vasculitis: 0–20%). In children with SLE, CSU was reported in 0–1.2% and CSU-like rash in 4.5–12% (urticarial vasculitis: 0–2.2%) (96). Specifically, significantly higher levels of IgE-anti-dsDNA, but not corresponding IgG levels, have also been found in a study of 85 chronic urticaria patients (97). Furthermore, basophils from 2 out of 9 of these patients exhibited degranulation in response to dsDNA, indicating functional relevance of these autoantibodies.

Beside these IgE antibodies that target antigens known in autoimmune diseases, patients with CSU can also develop IgE autoantibodies against probably disease-specific antigens. Schmetzer et al. found more than 200 IgE autoantigens in patients with CSU that were not found in healthy controls (98). Of the 31 IgE autoantigens detected in more than 70% of patients, 8 were soluble or membrane-bound and expressed in the skin and IgE autoantibodies to IL-24 were found in all patients with CSU. *In vitro* studies showed functional relevance, and clinically IgE-anti–IL-24 levels showed an association with disease activity. Detailed analysis of the remaining identified IgE targets had not been provided yet.

### Other Diseases

In many diseases, single or few reports have indicated a presence or potential role of autoreactive IgE.

#### Autoimmune Uveitis

Beside IgG autoantibodies, in autoimmune uveitis specific IgE to retinal S antigen was positive in 69% of 32 patients. In contrast, patients suffering from bacterial uveitis, as well as the healthy controls were negative for autoantibodies to retinal S antigen (29). Furthermore, IgE, IgG, and IgA anti-Galectin-1 (Gal-1) antibodies were increased in sera from patients with autoimmune uveitis and toxoplasmic retinochoroiditis compared to healthy controls (99). Both, anti-Gal-1 IgE and IgG antibodies were associated with progressive disease and poor disease outcome.

#### Rheumatoid Arthritis

A role of IgE antibodies in RA was first suggested several decades ago (27, 100). Antinuclear antibodies of the IgE class were found in 60% of 20 RA patients with neutropenia, whereas only 16% of RA patients without neutropenia had IgE antibodies of similar specificity (27). Anti-citrullinated protein antibodies are suggested to be highly specific and predictive for RA. In a study from Schuerwegh and colleagues, evidence for IgE directed against citrullinated protein was proposed (101), but later the paper was retracted (102). In recent times, no valid studies regarding this topic have been published.

#### Multiple Sclerosis

A role of IgE antibodies in the disease was suggested, despite the fact that there was no association between MS and allergies (103). In a study from Mikol and colleagues in 26 MS patients, a total of 128 peptides showed some IgE reactivity (mean 4.9 per subject), compared to 59 among the 15 controls (mean 3.9 per subject) (26). A detailed analysis of short, unique myelin protein-derived peptides (SUMPPs) revealed that for several SUMPPs, MS patients had significantly more reactive IgE, whereas for other SUMPPs, there was no significant difference between MS subjects and controls. The authors speculated that IgE reactive against CNS target antigens may have diagnostic and pathogenic significance.

#### Hyperthyroid Graves’ Disease

The presence of IgE autoantibodies in Grave’s disease was first proposed 40 years ago (104). Elevation of serum IgE ≥ 170 U/mL was found in up to 36% of the patients (105–108), but not in another study (109). Furthermore, there was immunohistochemical evidence for IgE involvement in Graves’ orbitopathy (110). Studies regarding specific IgE autoantibodies in thyroid disease showed IgE class TPO autoantibodies in 13 of 18 Graves’ and in 12 of 17 Hashimoto patients (24) and muscle autoantigens in thyroid associated ophthalmopathy (25). Evidence for a functional relevance of these IgE autoantibodies was shown by Raikow and colleagues, who showed that serum IgE is elevated in connection with certain stages of rapid dysthyroid orbitopathy progression (111).

#### Autoimmune Pancreatitis

Elevated total IgE levels are frequent in patients with autoimmune pancreatitis (21, 112), and it was recently suggested that analysis of total IgE in serum might be useful in the differentiation between autoimmune pancreatitis and pancreatic carcinoma (113). Nevertheless, IgE specific targets remain currently unknown, although there may be cross-reactivity with environmental antigens, as patients with higher IgE levels and with allergic diseases were more likely to have onset in March, April, May, August, September, or October (21).

#### Asthma

Autoallergic mechanisms have been proposed in murine models of asthma (17, 114). In human asthma, approximately 50% of patients with non-allergic asthma react to intradermal injection of autologous serum, indicating the presence of circulating vasoactive factors and suggesting the possibility of an autoreactive mechanism (18). In one patient with corticosteroid-dependent asthma associated with aspirin sensitivity, the presence of circulating IgE antibodies against 55 and 68 kDa platelet antigens has been described (115). Overall, autoallergy in asthma has not been studied in detail in humans so far.

#### Allergic Rhinoconjunctivitis

There is very limited evidence for IgE autoreactivity in allergic rhinoconjunctivitis. IgE antibodies from allergic individuals that bind to natural and recombinant birch profilin also bind to human profilin (16). Similar cross-reactivity was shown for human acidic ribosomal P2 protein in individuals sensitized to *Aspergillus fumigatus* P2 protein (116).

### Diseases in Which IgE Autoantibodies Were Looked for but Not Found

In chronic rhinosinusitis with nasal polyps no IgE autoreactivity to epithelial antigens, or anti-IgE IgG, has been detected, despite extensive tests (117).

## What do we know about the Relevance of IgE Autoantibodies in Different Diseases?

IgG-mediated autoimmune diseases occur in up to 9% of the population (118). Whether or not other immunoglobulin subtypes, including IgE, are critically involved in the pathogenesis of autoimmune diseases is largely unknown for most diseases. In contrast, polyreactive natural antibodies, which are not antigen-specific, can often detect self-antigens with low affinity. Most of these natural antibodies belong to the isotypes IgM, IgG_3_, and IgA, and these unspecific antibodies, even if they can bind to self-antigens, are thought to be protective, rather than harmful (119, 120). There is also some published evidence for the existence of natural IgE antibodies against pancreatic cancer cells (121) and conserved biotinylated-enzyme molecules present in many organisms, such as *Anisakis simplex, Toxocara canis, Ascaris suum*, and *Culex quinquefasciatus* (122, 123). While there are currently no data available on the role and relevance of natural IgE antibodies and how they could affect disease activity, there is some published evidence for a role of antigen-specific IgE against self in different diseases. For some diseases, there is only a single report on the potential role for auto-IgE. As reviewed above, in PV, high concentrations of serum IgE autoantibodies have been detected, and a strong correlation between dsg3-reactive IgE has been observed in patients with acute disease onset, indicating a role for auto-IgE in pemphigus (72). In patients with MS, there are some data indicating that IgE against CNS target antigens may have a pathogenic significance, particularly if other peptide-specific, potentially blocking immunoglobulins are absent (26). In other autoimmune diseases or diseases in which autoimmunity is thought to play a role, some more information on the potential role of auto-IgE has been published.

### Atopic Dermatitis

Although the pathogenesis of AD is still not completely understood, it is generally accepted that the underlying etiology is multifactorial, including environmental factors, impaired skin barrier function, and a hyper-immune activation (124). The role and relevance of autoimmunity in AD is as of yet unclear. In AD patients, many autoantigens can be detected, and IgE against self is highly prevalent (125, 126). Whether these auto-IgE antibodies indeed contribute to disease activity and severity or are merely bystanders cannot be concluded at present (125, 126). There is some evidence, however, that is suggestive of a pathogenic role for IgE against self in AD. Tang and colleagues have summarized this evidence and conclude that IgE autoreactivity is of importance in the pathogenesis of AD, especially because autoreactivity has been identified in various *in vitro* and direct clinical experiments across distinct study populations (19). Furthermore, significant associations between auto-IgE levels in AD patients and disease severity have been reported (37, 38), while a further two reports show a trend, but no significance for such a correlation (41, 46). In contrast, one investigation did not identify a correlation (39).

If auto-IgE is importantly involved in AD pathogenesis, treatment with anti-IgE antibodies should be able to improve symptoms. Because of the above mentioned investigations, omalizumab has been widely used in treatment refractory AD patients and many case reports and case series have been published. Two recent systematic reviews on the use of omalizumab in AD both come to the conclusion that anti-IgE treatment can be beneficial in the treatment of AD, but that larger clinical trials are missing to fully support this statement (127, 128).

### Systemic Lupus Erythematosus

Autoreactive IgE against a number of different antigens has been reported in SLE (see respective section above). In many investigations, a close correlation between levels of auto-IgG and auto-IgE in the same patients has been shown, making it difficult to estimate the contribution of auto-IgE to the pathology of SLE (49, 51, 129, 130). However, Henault and colleagues reported that the concentration of IgE anti-dsDNA correlates with disease severity and is likely to contribute to disease pathology independent from IgG anti-dsDNA (129). Dema and colleagues similarly show an association of the levels of IgE against different autoantibodies with SLE, comparable to autoreactive IgG levels (49). The best prediction of disease activity was found to be the presence of both, dsDNA-specific IgE and IgG, possibly indicating a role for auto-IgE in SLE (49).

In a recent publication, Pan and colleagues have shown that SLE patients not only have high levels of IgE directed against various autoantigens but also strongly decreased numbers of circulating basophils, suggesting auto-IgE-dependent basophil activation in the blood (131). These authors also stated that basophils from SLE patients showed high rates of activation that closely correlated with SLE activity. How this correlates with disease mechanisms is not clear. Anti-IgE treatment has not been reported in SLE patients as of yet.

#### Bullous Pemphigoid

The largest body of evidence for a pathological role of autoreactive IgE in the literature can be found for BP. In those patients presenting with specific IgE directed against the autoantigen BP180, a correlation of auto-IgE and disease severity has been reported (56, 60, 63). Moreover, Freire and colleagues have shown that IgE-anti-BP180 cannot only be found in the serum of BP patients, but also in the skin, bound to the surface of tissue-resident mast cells (132). Furthermore, they show that patient-derived IgE-anti-BP180 complexes can activate basophils, indicating the functionality of these antibodies (132).

In a number of case reports and case series, excellent efficacy of treatment with omalizumab has been shown in BP patients (133–140). While these findings are highly suggestive of a pathogenic role for auto-IgE in BP, controlled clinical trials with omalizumab in BP are necessary to prove the efficacy of anti-IgE treatment and thus of the relevance of auto-IgE in BP.

### Chronic Urticaria

The evidence for a pathogenetic role of auto-IgE in chronic urticaria has been extensively discussed in a recent review (141). This paper concluded that there are still many aspects of the pathologic mechanisms of CSU that need to be resolved, but that it is becoming increasingly clear that there are at least two distinct pathways, type I (IgE-mediated) and type II (IgG-mediated) autoimmunity, both of which contribute to the pathogenesis of this complex disease.

Since then, further evidence for a role for auto-IgE in CSU has been presented by Schmetzer and colleagues. Overall, more than 200 autoantigens that are recognized by IgE have been detected in CSU patients, and in 80% of more than 1,000 CSU patients, IgE-anti-IL-24 was present (98). Furthermore, levels of IgE-anti-IL-24 in the serum correlated with CSU disease activity, and *ex vivo*, IL-24 was able to activate mast cells after pre-incubation with serum from IgE-anti-IL-24 positive patients, indicating a biological relevance for IgE-anti-IL-24 in CSU patients (98).

The clinical relevance of IgE in both CSU and CindU has been proven in numerous case reports and case series and in a number of randomized-controlled trials using the anti-IgE antibody omalizumab (142–145). In fact, the first multi-center placebo-controlled study of omalizumab in CSU, the XCUISITE trial, was done in CSU patients who had IgE-anti-TPO (146). In this trial, 70% of patients of omalizumab-treated patients, but only 5% of placebo-treated patients experienced complete protection from wheal development, and the onset of omalizumab effects was early after the initiation of treatment. This strongly argues that the rapid and profound neutralization of auto-IgE by omalizumab contributed to its effects and that these IgE autoantibodies play a role in the pathogenesis of CSU. Further support for this comes from a recent study that showed that serum reactivity predicts the time to response to omalizumab therapy in CSU patients (147), suggesting that patients with IgG autoantibody-mediated CSU show delayed responses, whereas patients with IgE-autoantibody-mediated CSU have fast onset of improvement. The exact mechanisms by which anti-IgE treatment leads to the resolution of symptoms in most CSU patients are, as of yet, unclear. Nonetheless, at least in a proportion of CSU patients, the massive reduction of autoantigen-specific IgE is likely to be the most relevant mechanism.

In CindU, there is also a strong indication for an important role of IgE against self. For example, anti-IgE treatment has been shown to be highly effective in CindU in numerous case reports and case series, as well as in two randomized-controlled trials (Table 3). Furthermore, a direct involvement of IgE in some patients with CindU has been shown by passive transfer experiments. In cold urticaria (148, 149) as well as in symptomatic dermographism (150) disease activity was, in some patients, transferable from patients to healthy subjects by intradermal injection of patient sera. In these investigations, IgE was identified to be the relevant serum factor (148–150).

**Table 3:** Summary of chronic inducible urticaria patients showing a complete or partial response to omalizumab reported in case reports, case series, and clinical trials.

Condition	Total patients	No. of responders	Percentage responders
Symptomatic dermographism	58	43	74
Cold urticaria	51	39	76
Solar urticaria	47	34	72
Delayed pressure urticaria	32	29	91
Cholinergic urticaria	21	17	81
Heat urticaria	5	3	60
Vibratory angioedema	1	0	0
Aquagenic urticaria	1	1	100
Total	216	166	77

*Data contained in this table were obtained from Ref. (145, 151–153)*.

## What do we know about the Genetic Basis of IgE-Mediated Autoallergy

In an effort to understand the genetic influence on autoallergic diseases, it is best to look first at well-analyzed diseases such as SLE, where autoreactive IgE has been known for four decades (27). The prevalence of SLE and the presence of new autoreactive IgE are remarkably dependent on sex and race being 2.3-fold higher in Black persons than in White persons and 10-fold higher in females than in males (154). The greater incidence in females may be partially explained by the observation that women treated with estrogen and progesterone have a 1.34 times higher risk of developing SLE flares as compared to placebo treated.

Autoreactive IgE has been assessed in two SLE patient cohorts, IgE targeting classical SLE antigens being found in 63.3% of patients of French origin but only 52.9% of the patients from the United States (49). Most prominent in this study was IgE-anti-dsDNA but also auto-IgE against other SLE antigens (Sm, SSA/Ro, SSB/La). Non-SLE IgE only antigens (APEX, MPG, CLIP4) were also found. This autoreactive IgE correlated highly significantly with disease activity (SLEDAI score) and reduced C3 or C4 levels suggesting that IgE facilitates the amplification of autoimmune inflammation (155).

Chronic spontaneous urticaria is another example of an auto-IgE-mediated disease, where a strong and complex genetic bias *via* multiple alleles exists. These include *C5AR1* (156), *FPRL1* (157), *FcvarepsilonR1beta* (158), *TGFbeta1* (159, 160), IgE (161), IL-13 (162), histamine *N*-methyltransferase (163), *PTPN-22* (164, 165), *CTLA-4* (166), and *ACE* (167).

In addition to the well-known loci linked to hypersensitivity and atopy, such as *PLA2G7, MS4A2, IL4R* (168), *IL-10* (169), *IL12RB1* (170), *STAT4/6, CTLA-4* (171), and *GATA3* (172), recent studies identified many new alleles which regulate IgE production independent of external stimuli. However, these are often single findings, and only a genome-wide screening could weigh these findings and determine their relation to atopy, IgE and auto-IgE. One genome-wide screening on alleles influencing total IgE level identified *FCER1A* polymorphisms as having most impact, followed by *RAD50* and *STAT6* as the lowest relation (173).

One genetic variant has been studied in detail which links elevated IgE concentrations to a polymorphism in the *IL21R* gene promoter (174). This −83T–C promoter polymorphism in the *IL21R* gene is linked to a changed response of IgE synthesis to IFN-gamma stimulation (175). If *IL-21* is knocked-out in murine models, IgE production after immunization is increased, while IgG subtypes are decreased in otherwise normal animals (176). In contrast, loss-of-function of the *IL-21R* gene in humans leads to a severe primary immune defect (177). The difference to the murine model is not surprising, given the low degree of homology of the IgE system between man and rodent.

In addition to this *IL-21* gene variant, environmental receptor encoding genes, such as *NOD1* have been linked to asthma (178). This opens a crucial new viewpoint on genetics where environmental stimuli are related to selected genotypes to explain the phenotype. Another example is the β2-adrenoreceptor gene promoter polymorphism that determines modulation of IgE production as a response to xenobiotics (β2-agonists and glucocorticoids) (179).

These examples demonstrate the vast complexity of the genetic regulation of IgE synthesis (180). With modern deep sequencing strategies there are more and more variants found which change the risk for asthma and atopy, and influence IgE level. Sequencing of the IL4 gene alone lead to 14 new, previously unrecognized polymorphisms in addition to only two known previously (181). There are no studies on auto-IgE and *HLA* polymorphisms. However, IgE is involved in antigen uptake *via* immune complexes and presentation *via* MHC class II to CD4 cells (44, 182, 183). Therefore, there is a high likelihood that HLA genotypes will be linked to auto-IgE, but the studies need to be done.

## What do we know about the Cellular and Molecular Effects of IgE Autoantibodies?

When looking on the effects of IgE autoantibodies, mast cells and basophils are usually the focus of attention as they express high levels of the high-affinity receptor for IgE (FcεRI). Antigen-dependent activation of mast cells and basophils *via* FcεRI is the cause of acute allergic reactions induced by the rapid release of preformed mediators from granules and subsequent liberation of *de novo* synthesized lipids, cytokines, and chemokines. Yet, mast cells and basophils are not the only cells that express FcεRI and, importantly, other receptors such as CD23 (FcεRII) and galectin-3 are capable of binding IgE and inducing cellular responses. Thus, mast cell and basophil activation may be only the tip of the iceberg when considering the biology of IgE autoantibodies and their contribution to diseases. In particular, the function of CD23 in IgE-dependent sensitization of the host to autoallergens, which in turn may further strengthen the production of autoreactive IgE might be of great importance in the development of autoallergies.

In general, all IgE, whether it is autoreactive or whether it recognizes exogenous antigens, can trigger the same cellular responses as there is no evidence yet that autoreactive IgE have special molecular characteristics that drive certain functions/responses such as, for instance, cytokinergic activity. However, certain activities are not necessarily triggered by autoreactive IgE such as the CD23-mediated transepithelial transport of IgE. Here, we will focus on IgE-binding receptors with regards to their contribution in autoallergy.

### Effects of IgE/Autoantigen on FcεRI Signaling

The high-affinity receptor for IgE (FcεRI) consists of one IgE-binding α chain, which binds IgE with a very high affinity (10^10^ M^−1^), but does not contain intracellular signaling motives, one transmembrane-spanning β chain, which crosses the plasma membrane four times and contains an immunoreceptor tyrosine-based activation motive (ITAM), and two identical γ chains each containing an ITAM (184). This αβγγ motive is mainly expressed by mast cells and basophils although expression has also been shown in airway smooth muscle cells and bronchial epithelial cells of asthmatic patients (185, 186). A αγγ version of this receptor can be expressed by a variety of other cells including subsets of dendritic cells and monocytes/macrophages (187–189), eosinophils (190), neutrophils (191), and platelets (192, 193). Because of its high affinity for IgE, the receptor is occupied by monomeric IgE. When cross-linked by a multivalent antigen, it triggers signaling cascades leading to the immediate degranulation in mast cells and basophils, i.e., the release of preformed mediators from intracellular granules and *de novo* synthesis of lipid mediators such as prostaglandins and leukotrienes as well as the production of cytokines and chemokines and the expression of various surface markers. These include chemokine receptors such as CCR7 on plasmacytoid dendritic cells (pDCs) (129) or the selectin CD62L on basophils (194) that facilitate the migration of activated cells to local lymph nodes, but also co-stimulatory molecules like CD83 and CD86 on pDCs. Moreover, autoreactive IgE activates pDCs more efficiently and further synergizes with IgG through co-engagement of activating FcγRIIa, even in concentrations several orders of magnitude below IgG concentrations and drive B cell expansion and plasma call differentiation (129, 130). FcεRI engagement by IgE and allergen also induces IL-16 in Langerhans cells, a chemoattractant for CD4+ T cells, dendritic cells, and eosinophils (195). Thus, autoreactive IgE may have the capacity to augment disease activity in autoimmunity.

Surface bound IgE has been shown to stabilize and enhance the surface expression of FcεRI on mast cells and basophils, thereby allowing cells to bind more IgE, which in turn may lower the threshold for antigen-induced cell activation (196). This amplification loop might be of particular importance in autoallergic patients who present with increased serum and tissue IgE levels. Consistently, treatment with antibodies directed against IgE does not only lower serum IgE levels but also FcεRI surface expression on dendritic cells, basophils, and mast cells (197–199). Moreover, FcεRI-bound IgE promotes mast cell survival and migration (200). Interestingly, certain IgEs even induce mast cell and basophil degranulation and cytokine release in an antigen-independent manner (201). Although the *in vivo* relevance of this “cytokinergic” activity has not been demonstrated yet, it may have an impact in autoallergy. Cytokinergic IgE molecules associate with themselves and show an enhanced propensity to be polyreactive to various autoantigens (202, 203).

FcεRI-bound and antigen cross-linked IgE becomes internalized rapidly (129, 204). Thus, on the one hand, free serum IgE is cleared by dendritic cells and monocytes. On the other hand, when cross-linked, autoantigens become internalized and are capable of stimulating intracellular pattern recognition receptors such as TLRs. In pDCs, IgE autoantibodies directed against dsDNA become internalized and directed to phagolysosomes where TLR9 becomes activated to trigger the generation of inflammatory cytokines, such as INF-α, IL-6, IL-8, and TNF-α (129). Alternatively, internalized IgE/autoantigen is presented *via* MHC class-II by basophils, cutaneous Langerhans cells, or other dendritic cells in regional lymph nodes or local submucosal sites to naïve T cells to induce T_H_2 cells (194, 196, 205, 206). In systemic lupus erythematosus (SLE), activation of basophils by autoreactive IgE can induce the upregulation of CD62L, MHC-II and the B cell-activating factor BAFF. Activated basophils then migrate into the secondary lymphoid organs, where they produce IL-4 and IL-6, thereby promoting a T_H_2 environment with more IL-4 producing activated T cells and enhanced B cell proliferation with increased IgG_1_ and IgE production (194). In addition, CD1c+ dendritic cells may have the capacity for cross-presentation of internalized antigen to induce cytotoxic CD8+ cells. However, this is strongly inhibited by IL-4-producing T_H_2 cells (207). On monocytes, the engagement of FcεRI induces antibody-dependent cell-mediated cytotoxic activities of the cells rather than antibody-dependent cell-mediated phagocytic activities (ADCP), which are linked to the engagement of the low-affinity IgE receptor, CD23 (see below).

In BP, CSU and AD, the degranulation of mast cells and basophils contributes to disease manifestations. This, however, does not occur in systemic lupus erythematosus patients, who lack classical allergic manifestations. A possible mechanism for this is the co-engagement of inhibitory receptors such as FcγRIIb by autoreactive IgG in SLE (208). Moreover, cross-linking of FcεRI in monocytes and dendritic cells induces “late” anti-inflammatory IL-10, which can attenuate basophil and dendritic cell activation and suppresses monocyte phagocytic activity (209–211). Additionally, in monocytes, FcεRI engagement induces the upregulation of indoleamine 2,3-dioxygenase (IDO), which inhibits T cell proliferation and activates FOXP3+ regulatory T cells through the depletion of tryptophan (212).

A soluble version of FcεRI consisting of a single a-chain has been described (213). How sFcεRI is generated *in vivo* is not known so far. *In vitro* experiments suggest that it is generated during cellular FcεRI engagement. sFcεRI may bind to IgE with similar affinity as membrane-bound FcεRI and, therefore, might serve as a soluble regulator of free IgE and IgE-mediated cellular activation. Binding in a 1:1 ratio to IgE, it has the potential to prevent IgE from binding to the cellular receptor, and could negatively affect FceRI expression levels. *In vitro* and *in vivo* studies with sFcεRI show inhibitory properties on mast cell and basophil degranulation (214). Its impact in autoallergy, however, needs to be determined.

### Effects of IgE/Autoantigen on CD23 (FcεRII) Signaling

Most studies on the effects of IgE-mediated responses focus on mast cells or basophils and FcεRI signaling, as they are responsible for the immediate allergic response. However, the so-called “low affinity” receptor for IgE, CD23, has important functions in positively or negatively regulating IgE synthesis as well as antigen presentation to T cells (215). It is expressed on various cells including epithelial cells, activated B and T cells, Langerhans cells, plasma cells, monocytes, and eosinophils (196). CD23 belongs to the family of C-type lectins, which are calcium-dependent carbohydrate binding structures and contain three lectin domains located on the C-terminal extracellular head of the molecule. Interestingly, binding of IgE to the head domains seems not to involve carbohydrate structures (216). Although the affinity of a single CD23 head for the IgE-Fc is comparably low, the combined interaction of all three heads with IgE results in an strong binding with an affinity (10^8^–10^9^ M^−1^) that is comparable to that of FcεRI (215). Interestingly, CD23 is prone to shedding by disintegrin and metalloproteinase domain-containing protein 10 (ADAM10) resulting in various soluble forms (monomeric or trimeric with varying sizes), all of which are capable to bind IgE (214, 217) and are found in autoimmune diseases like SLE where auto-IgE has been described (218). Multiple ligands/interaction partners other than IgE have been described to interact with CD23 and are important for its function including the complement receptor 2, 3, and 4 (CD21; CD18/CD11b; CD18/CD11c), MHC-II, the receptor for vitronectin (αVβ3-integrin) and the αVβ5-integrin (215, 219). One important function of CD23 with regards to autoallergy is probably associated with the regulation of IgE production by B cells committed to IgE secretion. IgE-induced engagement of membrane CD23 induces a negative feedback while sCD23 (trimeric) through co-ligation of CD21 enhances IgE production (215). Moreover, CD23 bound IgE-antigen complexes can be presented to T cells *via* a process called facilitated antigen presentation (FAP). In FAP, the antigen-loaded IgE-CD23 gets internalized and loaded onto MHC-II molecules for presentation on the B-cells surface (219). A Th2 environment, as derived by activation of mast cells and basophils *via* FcεRII activation, may support this by increasing the expression of CD23 on B cells. Importantly, any activated B cell that expresses CD23, can present allergens to T cells independently of the specificity of the B cell receptor (BCR). By this, CD23-FAP can lead to epitope spreading, a process where the response to one epitope encountered by the BCR induces epitope-specific immunoglobulin production against the allergen internalized by CD23 (215). Epitope spreading can occur intra- or intermolecularly and is thought to be the driving cause for the development of polyspecific allergies to unrelated antigens. It is, therefore, of particular importance for the development of autoallergies. Antigen presentation via the CD23–MHC-II axis is as efficient as presentation of antigens by dendritic cells mediated by FcγRs and by far more efficient than BCR internalization (220). This is particularly dangerous when epitopes of an autoantigen and a pathogen are very similar in sequence and/or conformation (molecular mimicry) and IgE against the pathogen (IgE_PATH_) also binds to the autoantigen (221). Low-affinity IgE_PATH_-autoantigen interactions can lead *via* FAP dependent epitope spreading to high affinity auto-IgE. For many autoimmune diseases, such as SLE and BP, where IgE autoantibodies have been identified, B cell epitope spreading has been recognized as an important contributor for disease development.

On monocytes and macrophages, engagement of CD23 induces nitric oxide synthase and proinflammatory cytokines and importantly mediates IgE-dependent phagocytosis of targeted cells suggesting a use for immunotherapy, for instance in cancer (188, 222). Interestingly, auto-IgE to tumor antigens can be found in tumor tissue as well as systematically, suggesting a tumor suppressive function of these IgE autoantibodies.

### Effects of IgE/(Auto)Antigen on Galectin-3 Signaling

Galectin-3 is a secretory lectin containing a carbohydrate recognition domain connected to a non-lectin linker domain that associate to form a pentameric structure like IgM molecules. Galectin-3 is expressed by various immune cells including mast cells, basophils, neutrophils, monocytes, Langerhans cells, as well as T and B cells (196). It can be found in the nucleus, intracellular vesicles, or exosomes of expressing cells or gets secreted by a yet not fully characterized mechanism (223). Following secretion, it interacts with a large variety of cell surface and extracellular matrix proteins including IgE and FcεRI. Interestingly though, galectin-3 appears to have distinct binding capacities for IgE isoforms that are differentially glycosylated, although the consequence of this phenomenon in allergy and autoallergy is not clear (224). Because it is capable of binding to IgE and FcεRI, it can activate mast cells and other FcεRI-expressing and/or IgE-loaded cells in an IgE-dependent or independent manner and boost mast cell and basophil activation. In autoimmune diseases, galectin-3 is often increased in the serum and in tissues and may support auto-IgE-induced inflammation. However, most effects of galectin-3 on the immune system are independent of its ability to crosslink IgE and FcεRI. Its effects and relevance in autoallergy, therefore, need to be determined carefully (214, 225).

## Gaps of Knowledge, Unmet Needs, and Unanswered Questions

### Need for Improved Detection Methods

There are two different major drawbacks of the tests currently used to assess autoreactive IgE. First, different methods show a high variability and low reproducibility of IgE reactivity (226). This has been best demonstrated using the well characterized BP autoantigen BP180-NC16A. An improved ELISA method showed a significantly higher frequency of NC16A-specific IgE autoantibodies in the sera of BP patients than previously described (66). This study also demonstrated that most BP sera contain both, IgE and IgG class autoantibodies specific for NC16A and that IgG reactive to the same autoantigen as IgE can mask the detection of autoreactive IgE. This is the second major drawback of direct ELISA methods. This problem can be addressed by the use of indirect ELISA methods, where total IgE is first captured on the plate by anti-IgE and labeled autoantigen is used for detection (98). This approach, however, also has limitations. Saturation of the anti-IgE with non-autoreactive IgE in sera with very high total IgE concentrations can lead to the underestimation of autoallergen-specific IgE. Also, this method requires recombinant IgE to be able to calculate the specific IgE concentration in international units.

Yet unaddressed questions are the relevance of different conformational states of IgE, such as the bent-form, that may not be detectable by ELISA depending on which anti-IgE is used (227). Relevant cross-autoreactive IgE might also be prone to oligomerization due to stacking, which may hinder binding to capture antibodies.

Finally, autoreactive IgE may be preferentially captured in the tissue in patients with high FcεRI expression due to high IgE levels. Increased levels of IgE are frequently seen in patients with chronic urticaria and in atopic individuals (228, 229). *Vice versa*, as antigen reactivity encoded by the Fab-domain can change also the Fc part and, therefore, influence Fc receptor binding, some autoreactive IgEs might be over- or underrepresented in the non-cell bound IgE fraction due to changed FcεRI binding (230). Even assays detecting non-autoreactive IgE often fail to show a clinical relevance (231). The questions above need to be addressed for IgE in general, not only autoreactive IgE, in further studies, and improved methods need to be developed.

### What Leads to the Development of Autoantibodies of the IgE Isotype?

There are several possible mechanisms that might lead to a preference of IgE isotype for autoantibodies. Most IgE autoantigens are phosphorylated molecules (97, 232, 233). It has been demonstrated that IgG autoantibodies in BP preferably recognize a phosphorylated epitope (232, 234). The importance of phosphorylation for the development of autoantibodies in general has been shown for myeloperoxidase-anti-neutrophil cytoplasmic IgG autoantibodies in a mouse model (235). However, further studies are needed to assess whether this is a general rule preferably for IgE autoantigens, or for all autoantigens.

### IgE Cross-Reactivity Remains Largely Unexplained

In contrast to IgG and IgA, IgE has, in general, a much higher degree of cross-reactivity. This makes the determination of antigen-specific IgE difficult. As of now, very little is known about cross-reactivity of IgE autoantibodies. One study that performed site-directed mutagenesis of an allergenic peptide found that even after the mutation of all of the four residues that are mainly involved in IgE binding, the IgE still bound, albeit with a 100-fold reduced affinity (236). Furthermore, many studies have shown IgE cross-reactivity to structurally similar allergens, e.g., sensitization to the fungus *F. proliferatum* may lead to allergy to penicillin (237).

Usually, IgE cross-reactivity is explained by the recognition of carbohydrate-containing epitopes such as in cross-reactive IgEs against wheat/pollen or latex/hymenoptera proteins (238–241) or between different pollen allergens (242–244). Hierarchies of cross-reactivity toward different pollen allergens have been established (245).

In latex–pollen–food allergy, a good example of non-carbohydrate based cross-reactivity, cross-reactive IgE binds to latex and maize (246) or other food allergens (247). The antigenic epitopes of these allergens are well characterized and show only a low degree of sequence homology. The cross-reactivity can only be explained by a similar charge distribution, which, in the case of IgG, does usually not lead to cross-reactive antibodies (248).

Other examples of IgE cross-reactivity include pollen–food allergy, such as apple–birch allergy or pollen–fruit allergy (249–253), dog–cat allergy (254, 255), poultry–meat allergy (256), fish–chicken allergy (257), birch–oak allergy (258), latex–hymenoptera allergy (239–241). IgE cross reactivity to latex and parasites like *Schistosoma* has also been described (259, 260). In mice sensitized to birch pollen that also developed anaphylactic reactions to apple, desensitization to birch pollen also provided protection from anaphylaxis to apple (261). Similar experiences have been reported in allergic patients, where tolerance induction against one allergen also reduced reactivity toward other allergens (262). While IgE cross-reactivity between classical allergens is much more common than previously thought (263), cross-reactivity between autoallergens needs to be investigated in future studies.

### Unanswered Questions?

Many questions on IgE autoantibodies remain unanswered.

• Is IgE to self different from IgE to exogenous antigens in terms of its biochemical properties, its glycosylation, or its folding?
• Where is auto-IgE produced and what B cells are involved?
• Does the auto-IgE come from CD5+ B1 B cells of the marginal zone of the spleen (poorly negatively selected B cells that often produce autoantibodies) or from B cells that were previously allergen or pathogen reactive?
• Does the production of auto-IgE precede the onset of autoallergic signs and symptoms and when and why does it stop?

These and other questions are currently addressed by ongoing research. The answers that will come from these and future studies will help to better understand the biology and relevance of IgE autoantibodies in disease and to develop better approaches for the prevention and treatment of autoallergies.

## Author Contributions

All authors listed have made a substantial, direct, and intellectual contribution to the work and approved it for publication.

## Conflict of Interest Statement

The authors declare that the research was conducted in the absence of any commercial or financial relationships that could be construed as a potential conflict of interest.

## References

[1] Prausnitz C Kustner H Studien über die Ueberempfindlichkeit. Zentralbl Bakteriol (1921) 86:160–9.

[2] Ishizaka K Ishizaka T Identification of gamma-E-antibodies as a carrier of reaginic activity. J Immunol (1967) 99(6):1187–98. 4168663

[3] Johansson SG Bennich H Immunological studies of an atypical (myeloma) immunoglobulin. Immunology (1967) 13(4):381–94. 4168094 PMC1409218

[4] Johansson SG. The history of IgE: from discovery to 2010. Curr Allergy Asthma Rep (2011) 11(2):173–7. 10.1007/s11882-010-0174-3 21365369

[5] Marcolongo R Marsili C [Determination of serum IgD and IgE levels in patients with rheumatoid arthritis]. Reumatismo (1972) 24(2):173–4. 4681342

[6] Hunder GG Gleich GJ Immunoglobulin E (IgE) levels in serum and synovial fluid in rheumatoid arthritis. Arthritis Rheum (1974) 17(6):955–63. 10.1002/art.1780170606 4215429

[7] Marcolongo R Marsili C. Serum IgD and IgE in rheumatoid arthritis. Z Immunitatsforsch Exp Klin Immunol (1975) 148(4):285–90. 126543

[8] Arbesman CE Wypych JI Reisman RE Beutner EH IgE levels in sera of patients with pemphigus or bullous pemphigoid. Arch Dermatol (1974) 110(3):378–81. 10.1001/archderm.1974.01630090016003 4217592

[9] Provost TT Tomasi TB Jr Immunopathology of bullous pemphigoid. Basement membrane deposition of IgE, alternate pathway components and fibrin. Clin Exp Immunol (1974) 18(2):193–200. 4619597 PMC1537896

[10] Ogawa M Berger PA McIntyre OR Clendenning WE Ishizaka K IgE in atopic dermatitis. Arch Dermatol (1971) 103(6):575–80. 10.1001/archderm.1971.04000180001001 4104056

[11] Igarashi R [An immunohistochemical study of IgE in the skin of patients with systemic lupus erythematosus (author’s transl)]. Nihon Hifuka Gakkai Zasshi (1975) 85(7):385–93. 1081155

[12] Goldman JA Klimek GA Ali R. Allergy in systemic lupus erythematosus. IgE levels and reaginic phenomenon. Arthritis Rheum (1976) 19(4):669–76. 10.1002/1529-0131(197607/08)19:4<669::AID-ART1780190403>3.0.CO;2-E 942498

[13] Altrichter S Peter HJ Pisarevskaja D Metz M Martus P Maurer M IgE mediated autoallergy against thyroid peroxidase – a novel pathomechanism of chronic spontaneous urticaria? PLoS One (2011) 6(4):e14794 10.1371/journal.pone.0014794 21532759 PMC3075251

[14] Chang TW Chen C Lin CJ Metz M Church MK Maurer M. The potential pharmacologic mechanisms of omalizumab in patients with chronic spontaneous urticaria. J Allergy Clin Immunol (2015) 135(2):337–42. 10.1016/j.jaci.2014.04.036 24948369

[15] Metz M Ohanyan T Church MK Maurer M. Omalizumab is an effective and rapidly acting therapy in difficult-to-treat chronic urticaria: a retrospective clinical analysis. J Dermatol Sci (2014) 73(1):57–62. 10.1016/j.jdermsci.2013.08.011 24060603

[16] Valenta R Duchene M Pettenburger K Sillaber C Valent P Bettelheim P Identification of profilin as a novel pollen allergen; IgE autoreactivity in sensitized individuals. Science (1991) 253(5019):557–60. 10.1126/science.1857985 1857985

[17] Garn H Mittermann I Valenta R Renz H. Autosensitization as a pathomechanism in asthma. Ann N Y Acad Sci (2007) 1107:417–25. 10.1196/annals.1381.044 17804570

[18] Tedeschi A Asero R. Asthma and autoimmunity: a complex but intriguing relation. Expert Rev Clin Immunol (2008) 4(6):767–76. 10.1586/1744666X.4.6.767 20477126

[19] Tang TS Bieber T Williams HC Does “autoreactivity” play a role in atopic dermatitis? J Allergy Clin Immunol (2012) 129(5):1209–15.e2. 10.1016/j.jaci.2012.02.002 22409986

[20] Valenta R Seiberler S Natter S Mahler V Mossabeb R Ring J Autoallergy: a pathogenetic factor in atopic dermatitis? J Allergy Clin Immunol (2000) 105(3):432–7. 10.1067/mai.2000.104783 10719290

[21] Zhang L Guo L Huang Y Wang T Shi X Chang H Allergic diseases, immunoglobulin E, and autoimmune pancreatitis: a retrospective study of 22 patients. Chin Med J (Engl) (2014) 127(23):4104–9. 25430457

[22] van Beek N Schulze FS Zillikens D Schmidt E. IgE-mediated mechanisms in bullous pemphigoid and other autoimmune bullous diseases. Expert Rev Clin Immunol (2016) 12(3):267–77. 10.1586/1744666X.2016.1123092 26588556

[23] Panaszek B Pawlowicz R Grzegrzolka J Obojski A. Autoreactive IgE in chronic spontaneous/idiopathic urticaria and basophil/mastocyte priming phenomenon, as a feature of autoimmune nature of the syndrome. Arch Immunol Ther Exp (Warsz) (2017) 65(2):137–43. 10.1007/s00005-016-0417-7 27582030

[24] Guo J Rapoport B McLachlan SM. Thyroid peroxidase autoantibodies of IgE class in thyroid autoimmunity. Clin Immunol Immunopathol (1997) 82(2):157–62. 10.1006/clin.1996.4297 9000484

[25] Elisei R Weightman D Kendall-Taylor P Vassart G Ludgate M. Muscle autoantigens in thyroid associated ophthalmopathy: the limits of molecular genetics. J Endocrinol Invest (1993) 16(7):533–40. 10.1007/BF03348900 8227983

[26] Mikol DD Ditlow C Usatin D Biswas P Kalbfleisch J Milner A Serum IgE reactive against small myelin protein-derived peptides is increased in multiple sclerosis patients. J Neuroimmunol (2006) 180(1–2):40–9. 10.1016/j.jneuroim.2006.06.030 16996143

[27] Permin H Wiik A. The prevalence of IgE antinuclear antibodies in rheumatoid arthritis and systemic lupus erythematosus. Acta Pathol Microbiol Scand C (1978) 86C(5):245–9. 309705 10.1111/j.1699-0463.1978.tb02587.x

[28] Sanjuan MA Sagar D Kolbeck R. Role of IgE in autoimmunity. J Allergy Clin Immunol (2016) 137(6):1651–61. 10.1016/j.jaci.2016.04.007 27264000

[29] Muino JC Juarez CP Luna JD Castro CC Wolff EG Ferrero M The importance of specific IgG and IgE autoantibodies to retinal S antigen, total serum IgE, and sCD23 levels in autoimmune and infectious uveitis. J Clin Immunol (1999) 19(4):215–22. 10.1023/A:1020516029883 10471975

[30] Keller P Beitrag zu den beziehungen von asthma und ekzem. Arch Derm Syph Berl (1924) 148:82–91. 10.1007/BF01827500

[31] Storm van Leeuwen W Bien Z Varekamp H Über die hautreaktion mit extrakten menschlicher kopfhautschuppen bei allergischen krankheiten. Klin Wochenschr (1926) 5:1023–5. 10.1007/BF01717944

[32] Hampton SF Cooke RA The sensitivity of man to human dander, with particular reference to eczema (allergic dermatitis). J Allergy (1941) 13:63–76. 10.1016/S0021-8707(41)90008-4

[33] Simon FA Human dander: an important cause of infantile eczema. JAMA (1944) 125:350–9. 10.1001/jama.1944.02850230030008

[34] Simon FA On the allergen in human dander. J Allergy (1944) 15:338–45. 10.1016/S0021-8707(44)90143-7

[35] Simon FA The allergen of human dander present in skin of the general body surface. J Invest Dermatol (1947) 9(6):329–32. 10.1038/jid.1947.106 18918928

[36] Valenta R Maurer D Steiner R Seiberler S Sperr WR Valent P Immunoglobulin E response to human proteins in atopic patients. J Invest Dermatol (1996) 107(2):203–8. 10.1111/1523-1747.ep12329617 8757763

[37] Szakos E Lakos G Aleksza M Gyimesi E Pall G Fodor B Association between the occurrence of the anticardiolipin IgM and mite allergen-specific IgE antibodies in children with extrinsic type of atopic eczema/dermatitis syndrome. Allergy (2004) 59(2):164–7. 10.1046/j.1398-9995.2003.00367.x 14763929

[38] Schmid-Grendelmeier P Fluckiger S Disch R Trautmann A Wuthrich B Blaser K IgE-mediated and T cell-mediated autoimmunity against manganese superoxide dismutase in atopic dermatitis. J Allergy Clin Immunol (2005) 115(5):1068–75. 10.1016/j.jaci.2005.01.065 15867868

[39] Higashi N Niimi Y Aoki M Kawana S. Clinical features of antinuclear antibody-positive patients with atopic dermatitis. J Nippon Med Sch (2009) 76(6):300–7. 10.1272/jnms.76.300 20035096

[40] Altrichter S Kriehuber E Moser J Valenta R Kopp T Stingl G. Serum IgE autoantibodies target keratinocytes in patients with atopic dermatitis. J Invest Dermatol (2008) 128(9):2232–9. 10.1038/jid.2008.80 18480840

[41] Zeller S Rhyner C Meyer N Schmid-Grendelmeier P Akdis CA Crameri R. Exploring the repertoire of IgE-binding self-antigens associated with atopic eczema. J Allergy Clin Immunol (2009) 124(2): 278–85, 285.e1–7. 10.1016/j.jaci.2009.05.015 19541355

[42] Balaji H Heratizadeh A Wichmann K Niebuhr M Crameri R Scheynius A *Malassezia sympodialis* thioredoxin-specific T cells are highly cross-reactive to human thioredoxin in atopic dermatitis. J Allergy Clin Immunol (2011) 128(1):92–9.e4. 10.1016/j.jaci.2011.02.043 21489611

[43] Watanabe K Muro Y Sugiura K Tomita Y. IgE and IgG(4) autoantibodies against DFS70/LEDGF in atopic dermatitis. Autoimmunity (2011) 44(6):511–9. 10.3109/08916934.2010.549157 21329475

[44] Natter S Seiberler S Hufnagl P Binder BR Hirschl AM Ring J Isolation of cDNA clones coding for IgE autoantigens with serum IgE from atopic dermatitis patients. FASEB J (1998) 12(14):1559–69. 10.1096/fasebj.12.14.1559 9806765

[45] Valenta R Natter S Seiberler S Wichlas S Maurer D Hess M Molecular characterization of an autoallergen, Hom s 1, identified by serum IgE from atopic dermatitis patients. J Invest Dermatol (1998) 111(6):1178–83. 10.1046/j.1523-1747.1998.00413.x 9856836

[46] Mothes N Niggemann B Jenneck C Hagemann T Weidinger S Bieber T The cradle of IgE autoreactivity in atopic eczema lies in early infancy. J Allergy Clin Immunol (2005) 116(3):706–9. 10.1016/j.jaci.2005.06.025 16159648

[47] Zhu H Luo H Yan M Zuo X Li QZ. Autoantigen microarray for high-throughput autoantibody profiling in systemic lupus erythematosus. Genomics Proteomics Bioinformatics (2015) 13(4):210–8. 10.1016/j.gpb.2015.09.001 26415621 PMC4610965

[48] Atta AM Santiago MB Guerra FG Pereira MM Sousa Atta ML. Autoimmune response of IgE antibodies to cellular self-antigens in systemic lupus erythematosus. Int Arch Allergy Immunol (2010) 152(4):401–6. 10.1159/000288293 20197682

[49] Dema B Pellefigues C Hasni S Gault N Jiang C Ricks TK Autoreactive IgE is prevalent in systemic lupus erythematosus and is associated with increased disease activity and nephritis. PLoS One (2014) 9(2):e90424 10.1371/journal.pone.0090424 24587356 PMC3938730

[50] Hanaoka H Okazaki Y Satoh T Kaneko Y Yasuoka H Seta N Circulating anti-double-stranded DNA antibody-secreting cells in patients with systemic lupus erythematosus: a novel biomarker for disease activity. Lupus (2012) 21(12):1284–93. 10.1177/0961203312453191 22740429

[51] Rhyner C Daigle I Crameri R Auto-reactive IgE responses to acidic ribosomal P(2) protein in systemic lupus erythematosus. Allergy (2011) 66(8):1127–9. 10.1111/j.1398-9995.2011.02581.x 21410706

[52] Sekigawa I Seta N Yamada M Iida N Hashimoto H Ogawa H. Possible importance of immunoglobulin E in foetal loss by mothers with anti-SSA antibody. Scand J Rheumatol (2004) 33(1):44–6. 10.1080/03009740310004658 15124942

[53] Brucato A Cimaz R Caporali R Ramoni V Buyon J. Pregnancy outcomes in patients with autoimmune diseases and anti-Ro/SSA antibodies. Clin Rev Allergy Immunol (2011) 40(1):27–41. 10.1007/s12016-009-8190-6 20012231 PMC3558034

[54] Christophoridis S Budinger L Borradori L Hunziker T Merk HF Hertl M. IgG, IgA and IgE autoantibodies against the ectodomain of BP180 in patients with bullous and cicatricial pemphigoid and linear IgA bullous dermatosis. Br J Dermatol (2000) 143(2):349–55. 10.1046/j.1365-2133.2000.03661.x 10951144

[55] Thoma-Uszynski S Uter W Schwietzke S Hofmann SC Hunziker T Bernard P BP230- and BP180-specific auto-antibodies in bullous pemphigoid. J Invest Dermatol (2004) 122(6):1413–22. 10.1111/j.0022-202X.2004.22603.x 15175032

[56] van Beek N Luttmann N Huebner F Recke A Karl I Schulze FS Correlation of serum levels of IgE autoantibodies against BP180 with bullous pemphigoid disease activity. JAMA Dermatol (2017) 153(1):30–8. 10.1001/jamadermatol.2016.3357 27829102

[57] Hofmann S Thoma-Uszynski S Hunziker T Bernard P Koebnick C Stauber A Severity and phenotype of bullous pemphigoid relate to autoantibody profile against the NH2- and COOH-terminal regions of the BP180 ectodomain. J Invest Dermatol (2002) 119(5):1065–73. 10.1046/j.1523-1747.2002.19529.x 12445194

[58] Dresow SK Sitaru C Recke A Oostingh GJ Zillikens D Gibbs BF. IgE autoantibodies against the intracellular domain of BP180. Br J Dermatol (2009) 160(2):429–32. 10.1111/j.1365-2133.2008.08858.x 18808416

[59] Dimson OG Giudice GJ Fu CL Van den Bergh F Warren SJ Janson MM Identification of a potential effector function for IgE autoantibodies in the organ-specific autoimmune disease bullous pemphigoid. J Invest Dermatol (2003) 120(5):784–8. 10.1046/j.1523-1747.2003.12146.x 12713582

[60] Iwata Y Komura K Kodera M Usuda T Yokoyama Y Hara T Correlation of IgE autoantibody to BP180 with a severe form of bullous pemphigoid. Arch Dermatol (2008) 144(1):41–8. 10.1001/archdermatol.2007.9 18209167

[61] Ishiura N Fujimoto M Watanabe R Nakashima H Kuwano Y Yazawa N Serum levels of IgE anti-BP180 and anti-BP230 autoantibodies in patients with bullous pemphigoid. J Dermatol Sci (2008) 49(2):153–61. 10.1016/j.jdermsci.2007.08.008 17920818

[62] Yayli S Pelivani N Beltraminelli H Wirthmuller U Beleznay Z Horn M Detection of linear IgE deposits in bullous pemphigoid and mucous membrane pemphigoid: a useful clue for diagnosis. Br J Dermatol (2011) 165(5):1133–7. 10.1111/j.1365-2133.2011.10481.x 21711326

[63] Dopp R Schmidt E Chimanovitch I Leverkus M Brocker EB Zillikens D. IgG4 and IgE are the major immunoglobulins targeting the NC16A domain of BP180 in bullous pemphigoid: serum levels of these immunoglobulins reflect disease activity. J Am Acad Dermatol (2000) 42(4):577–83. 10.1067/mjd.2000.103986 10727301

[64] Pomponi D Di Zenzo G Zennaro D Calabresi V Eming R Zuzzi S Detection of IgG and IgE reactivity to BP180 using the ISAC(R) microarray system. Br J Dermatol (2013) 168(6):1205–14. 10.1111/bjd.12161 23252883

[65] Liu B Zuo YG Zhou XP He CX Li J Tie D [Establishment of enzyme-linked immunosorbent assay in the detection of BP180NC16A-specific IgE and its significance in bullous pemphigoid]. Zhonghua Yi Xue Za Zhi (2013) 93(28):2244–7. 24169339

[66] Messingham KA Noe MH Chapman MA Giudice GJ Fairley JA. A novel ELISA reveals high frequencies of BP180-specific IgE production in bullous pemphigoid. J Immunol Methods (2009) 346(1–2):18–25. 10.1016/j.jim.2009.04.013 19422829 PMC2703696

[67] Engineer L Bhol K Kumari S Razzaque Ahmed A. Bullous pemphigoid: interaction of interleukin 5, anti-basement membrane zone antibodies and eosinophils. A preliminary observation. Cytokine (2001) 13(1):32–8. 10.1006/cyto.2000.0791 11145840

[68] Fania L Caldarola G Muller R Brandt O Pellicano R Feliciani C IgE recognition of bullous pemphigoid (BP)180 and BP230 in BP patients and elderly individuals with pruritic dermatoses. Clin Immunol (2012) 143(3):236–45. 10.1016/j.clim.2012.02.003 22534318

[69] Cozzani E Micalizzi C Parodi A Rebora A Anti-230 kDa circulating IgE in bullous pemphigoid: relationship with disease activity. Acta Derm Venereol (1997) 77(3):236. 9188881 10.2340/0001555577236

[70] Ghohestani RF Cozzani E Delaporte E Nicolas JF Parodi A Claudy A. IgE antibodies in sera from patients with bullous pemphigoid are autoantibodies preferentially directed against the 230-kDa epidermal antigen (BP230). J Clin Immunol (1998) 18(3):202–9. 10.1023/A:1020531005776 9624579

[71] Bruns GR Ablin RJ Guinan PD. Serum immunoglobulin E in pemphigus. J Invest Dermatol (1978) 71(3):217–8. 10.1111/1523-1747.ep12547283 690486

[72] Nagel A Lang A Engel D Podstawa E Hunzelmann N de Pita O Clinical activity of pemphigus vulgaris relates to IgE autoantibodies against desmoglein 3. Clin Immunol (2010) 134(3):320–30. 10.1016/j.clim.2009.11.006 20015693

[73] Spaeth S Riechers R Borradori L Zillikens D Budinger L Hertl M. IgG, IgA and IgE autoantibodies against the ectodomain of desmoglein 3 in active pemphigus vulgaris. Br J Dermatol (2001) 144(6):1183–8. 10.1046/j.1365-2133.2001.04228.x 11422039

[74] Natsuga K Nishie W Shinkuma S Moriuchi R Shibata M Nishimura M Circulating IgA and IgE autoantibodies in antilaminin-332 mucous membrane pemphigoid. Br J Dermatol (2010) 162(3):513–7. 10.1111/j.1365-2133.2009.09508.x 19751242

[75] Qian Y Prisayanh P Andraca E Qaqish BF Aoki V Hans-Filhio G IgE, IgM, and IgG4 anti-desmoglein 1 autoantibody profile in endemic pemphigus foliaceus (fogo selvagem). J Invest Dermatol (2011) 131(4):985–7. 10.1038/jid.2010.403 21191415 PMC3164298

[76] Qian Y Jeong JS Abdeladhim M Valenzuela JG Aoki V Hans-Filhio G IgE anti-LJM11 sand fly salivary antigen may herald the onset of fogo selvagem in endemic Brazilian regions. J Invest Dermatol (2015) 135(3):913–5. 10.1038/jid.2014.430 25285921 PMC4323842

[77] Maurer M Metz M Magerl M Siebenhaar F Staubach P [Autoreactive urticaria and autoimmune urticaria]. Hautarzt (2004) 55(4):350–6. 10.1007/s00105-004-0692-9 14999391

[78] Maurer M Rosen K Hsieh HJ Saini S Grattan C Gimenez-Arnau A Omalizumab for the treatment of chronic idiopathic or spontaneous urticaria. N Engl J Med (2013) 368(10):924–35. 10.1056/NEJMoa1215372 23432142

[79] Caliskaner Z Ozturk S Turan M Karaayvaz M. Skin test positivity to aeroallergens in the patients with chronic urticaria without allergic respiratory disease. J Investig Allergol Clin Immunol (2004) 14(1):50–4. 15160442

[80] Kulthanan K Jiamton S Rutnin NO Insawang M Pinkaew S. Prevalence and relevance of the positivity of skin prick testing in patients with chronic urticaria. J Dermatol (2008) 35(6):330–5. 10.1111/j.1346-8138.2008.00477.x 18578709

[81] Gecer E Erdem T Aeroallergen prick skin test and autologous serum skin test results in patients with chronic urticaria and their comparison. Ann Dermatol (2012) 24(4):472–4. 10.5021/ad.2012.24.4.472 23197917 PMC3505782

[82] Song Z Zhai Z Zhong H Zhou Z Chen W Hao F. Evaluation of autologous serum skin test and skin prick test reactivity to house dust mite in patients with chronic spontaneous urticaria. PLoS One (2013) 8(5):e64142. 10.1371/journal.pone.0064142 23741306 PMC3669345

[83] Staubach P Vonend A Burow G Metz M Magerl M Maurer M. Patients with chronic urticaria exhibit increased rates of sensitisation to *Candida albicans*, but not to common moulds. Mycoses (2009) 52(4):334–8. 10.1111/j.1439-0507.2008.01601.x 18793264

[84] Zhang M Liu F Liu H Shen Y Kong Q Sang H. Sensitization and cross-reactions of dermatophyte and *Candida albicans* allergens in patients with chronic urticaria. Int J Dermatol (2016) 55(10):1138–42. 10.1111/ijd.13162 27420771

[85] Kulthanan K Wachirakaphan C. Prevalence and clinical characteristics of chronic urticaria and positive skin prick testing to mites. Acta Derm Venereol (2008) 88(6):584–8. 10.2340/00015555-0546 19002343

[86] Zuberbier T Balke M Worm M Edenharter G Maurer M. Epidemiology of urticaria: a representative cross-sectional population survey. Clin Exp Dermatol (2010) 35(8):869–73. 10.1111/j.1365-2230.2010.03840.x 20456386

[87] Augey F Gunera-Saad N Bensaid B Nosbaum A Berard F Nicolas JF Chronic spontaneous urticaria is not an allergic disease. Eur J Dermatol (2011) 21(3):349–53. 10.1684/ejd.2011.1285 21616748

[88] Kolkhir P Metz M Altrichter S Maurer M Comorbidity of chronic spontaneous urticaria and autoimmune thyroid diseases: a systematic review. Allergy (2017) 72(10):1440–60. 10.1111/all.13182 28407273

[89] Bar-Sela S Reshef T Mekori YA IgE antithyroid microsomal antibodies in a patient with chronic urticaria. J Allergy Clin Immunol (1999) 103(6):1216–7. 10.1016/S0091-6749(99)70204-6 10359911

[90] Atta AM Rodrigues MZ Sousa CP Medeiros Junior M Sousa-Atta ML Autoantibody production in chronic idiopathic urticaria is not associated with *Helicobacter pylori* infection. Braz J Med Biol Res (2004) 37(1):13–7. 10.1590/S0100-879X2004000100002 14689038

[91] Concha LB Chang CC Szema AM Dattwyler RJ Carlson HE. IgE antithyroid antibodies in patients with Hashimoto’s disease and chronic urticaria. Allergy Asthma Proc (2004) 25(5):293–6. 15603201

[92] Tedeschi A Lorini M Asero R Anti-thyroid peroxidase IgE in patients with chronic urticaria. J Allergy Clin Immunol (2001) 108(3):467–8. 10.1067/mai.2001.117792 11544471

[93] Kadooka Y Idota T Gunji H Shimatani M Kawakami H Dosako S A method for measuring specific IgE in sera by direct ELISA without interference by IgG competition or IgG autoantibodies to IgE. Int Arch Allergy Immunol (2000) 122(4):264–9. 10.1159/000024408 10971117

[94] Shin YS Suh DH Yang EM Ye YM Park HS. Serum specific IgE to thyroid peroxidase activates basophils in aspirin intolerant urticaria. J Korean Med Sci (2015) 30(6):705–9. 10.3346/jkms.2015.30.6.705 26028921 PMC4444469

[95] Kolkhir P Borzova E Grattan C Asero R Pogorelov D Maurer M Autoimmune comorbidity in chronic spontaneous urticaria: a systematic review. Autoimmun Rev (2017) 16(12):1196–208. 10.1016/j.autrev.2017.10.003 29037900

[96] Kolkhir P Pogorelov D Olisova O Maurer M Comorbidity and pathogenic links of chronic spontaneous urticaria and systemic lupus erythematosus – a systematic review. Clin Exp Allergy (2016) 46(2):275–87. 10.1111/cea.12673 26545308

[97] Hatada Y Kashiwakura J Hayama K Fujisawa D Sasaki-Sakamoto T Terui T Significantly high levels of anti-dsDNA immunoglobulin E in sera and the ability of dsDNA to induce the degranulation of basophils from chronic urticaria patients. Int Arch Allergy Immunol (2013) 161(Suppl 2):154–8. 10.1159/000350388 23711867

[98] Schmetzer O Lakin E Topal FA Preusse P Freier D Church MK IL-24 is a common and specific autoantigen of IgE in patients with chronic spontaneous urticaria. J Allergy Clin Immunol (2017). 10.1016/j.jaci.2017.10.035 29208545

[99] Romero MD Muino JC Bianco GA Ferrero M Juarez CP Luna JD Circulating anti-galectin-1 antibodies are associated with the severity of ocular disease in autoimmune and infectious uveitis. Invest Ophthalmol Vis Sci (2006) 47(4):1550–6. 10.1167/iovs.05-1234 16565391

[100] Meretey K Falus A Erhardt CC Maini RN. IgE and IgE-rheumatoid factors in circulating immune complexes in rheumatoid arthritis. Ann Rheum Dis (1982) 41(4):405–8. 10.1136/ard.41.4.405 6981388 PMC1000959

[101] Schuerwegh AJ Ioan-Facsinay A Dorjee AL Roos J Bajema IM van der Voort EI Evidence for a functional role of IgE anticitrullinated protein antibodies in rheumatoid arthritis. Proc Natl Acad Sci U S A (2010) 107(6):2586–91. 10.1073/pnas.0913054107 20133791 PMC2823893

[102] Schuerwegh AJ Ioan-Facsinay A Dorjee AL Roos J Bajema IM van der Voort EI Retraction for Schuerwegh et al., evidence for a functional role of IgE anticitrullinated protein antibodies in rheumatoid arthritis. Proc Natl Acad Sci U S A (2013) 110(50):20345 10.1073/pnas.1320459110 24277813 PMC3864332

[103] Monteiro L Souza-Machado A Menezes C Melo A. Association between allergies and multiple sclerosis: a systematic review and meta-analysis. Acta Neurol Scand (2011) 123(1):1–7. 10.1111/j.1600-0404.2010.01355.x 20456246

[104] Matsui Y Heiner DC Beall GN IgE and IgE autoantibodies in patients with autoimmune thyroid disorders and their relatives. Proc Soc Exp Biol Med (1978) 158(1):73–6. 10.3181/00379727-158-40142 580805

[105] Inoue M Rakugi H Nakamaru M Masugi F Ogihara T Takai S. [Graves’ disease with markedly elevated serum immunoglobulin E]. Nihon Naibunpi Gakkai Zasshi (1989) 65(11):1264–9. 2591610 10.1507/endocrine1927.65.11_1264

[106] Sayinalp S Akalin S Sayinalp N Erbas T Bayraktar M Ozcebe OI Serum immunoglobulin E and soluble CD23 in patients with Graves’ disease. Horm Metab Res (1996) 28(3):133–7. 10.1055/s-2007-979145 8926012

[107] Sato A Takemura Y Yamada T Ohtsuka H Sakai H Miyahara Y A possible role of immunoglobulin E in patients with hyperthyroid Graves’ disease. J Clin Endocrinol Metab (1999) 84(10):3602–5. 10.1210/jcem.84.10.6038 10523002

[108] Yamada T Sato A Komiya I Nishimori T Ito Y Terao A An elevation of serum immunoglobulin E provides a new aspect of hyperthyroid Graves’ disease. J Clin Endocrinol Metab (2000) 85(8):2775–8. 10.1210/jcem.85.8.6741 10946880

[109] Latifi-Pupovci H Gacaferri-Lumezi B Lokaj-Berisha V. There is no elevation of immunoglobulin e levels in Albanian patients with autoimmune thyroid diseases. J Thyroid Res (2014) 2014:283709. 10.1155/2014/283709 24959371 PMC4053298

[110] Raikow RB Dalbow MH Kennerdell JS Compher K Machen L Hiller W Immunohistochemical evidence for IgE involvement in Graves’ orbitopathy. Ophthalmology (1990) 97(5):629–35. 10.1016/S0161-6420(90)32548-4 2188194

[111] Raikow RB Tyutyunikov A Kennerdell JS Kazim M Dalbow MH Scalise D. Correlation of serum immunoglobulin E elevations with clinical stages of dysthyroid orbitopathy. Ophthalmology (1992) 99(3):361–5. 10.1016/S0161-6420(92)31964-5 1565448

[112] Hirano K Tada M Isayama H Kawakubo K Yagioka H Sasaki T Clinical analysis of high serum IgE in autoimmune pancreatitis. World J Gastroenterol (2010) 16(41):5241–6. 10.3748/wjg.v16.i41.5241 21049558 PMC2975095

[113] van Toorenenbergen AW van Heerde MJ van Buuren HR. Potential value of serum total IgE for differentiation between autoimmune pancreatitis and pancreatic cancer. Scand J Immunol (2010) 72(5):444–8. 10.1111/j.1365-3083.2010.02453.x 21039739

[114] Bunder R Mittermann I Herz U Focke M Wegmann M Valenta R Induction of autoallergy with an environmental allergen mimicking a self protein in a murine model of experimental allergic asthma. J Allergy Clin Immunol (2004) 114(2):422–8. 10.1016/j.jaci.2004.05.029 15316527

[115] Lassalle P Joseph M Ramon P Dracon M Tonnel AB Capron A. Plasmapheresis in a patient with severe asthma associated with auto-antibodies to platelets. Clin Exp Allergy (1990) 20(6):707–12. 10.1111/j.1365-2222.1990.tb02712.x 2083410

[116] Mayer C Appenzeller U Seelbach H Achatz G Oberkofler H Breitenbach M Humoral and cell-mediated autoimmune reactions to human acidic ribosomal P2 protein in individuals sensitized to *Aspergillus fumigatus* P2 protein. J Exp Med (1999) 189(9):1507–12. 10.1084/jem.189.9.1507 10224291 PMC2193053

[117] De Schryver E Calus L Bonte H Natalie R Gould H Donovan E The quest for autoreactive antibodies in nasal polyps. J Allergy Clin Immunol (2016) 138(3):893–895.e5. 10.1016/j.jaci.2016.03.040 27283383 PMC5514560

[118] Cooper GS Bynum ML Somers EC. Recent insights in the epidemiology of autoimmune diseases: improved prevalence estimates and understanding of clustering of diseases. J Autoimmun (2009) 33(3–4):197–207. 10.1016/j.jaut.2009.09.008 19819109 PMC2783422

[119] Panda S Ding JL. Natural antibodies bridge innate and adaptive immunity. J Immunol (2015) 194(1):13–20. 10.4049/jimmunol.1400844 25527792

[120] Kapsogeorgou EK Tzioufas AG. Autoantibodies in autoimmune diseases: clinical and Critical evaluation. Isr Med Assoc J (2016) 18(9):519–24. 28471596

[121] Fu SL Pierre J Smith-Norowitz TA Hagler M Bowne W Pincus MR Immunoglobulin E antibodies from pancreatic cancer patients mediate antibody-dependent cell-mediated cytotoxicity against pancreatic cancer cells. Clin Exp Immunol (2008) 153(3):401–9. 10.1111/j.1365-2249.2008.03726.x 18803764 PMC2527370

[122] Das MK Mishra A Beuria MK Dash AP. Human natural antibodies to *Culex quinquefasciatus*: age-dependent occurrence. J Am Mosq Control Assoc (1991) 7(2):319–21. 1895094

[123] Lorenzo S Iglesias R Paniagua E Ansotegui I Alonso JM Ubeira FM. Natural antibodies to nematode biotinyl-enzymes in human sera. Med Microbiol Immunol (2001) 189(4):177–83. 10.1007/s004300100065 11599787

[124] Otsuka A Nomura T Rerknimitr P Seidel JA Honda T Kabashima K. The interplay between genetic and environmental factors in the pathogenesis of atopic dermatitis. Immunol Rev (2017) 278(1):246–62. 10.1111/imr.12545 28658541

[125] Cipriani F Ricci G Leoni MC Capra L Baviera G Longo G Autoimmunity in atopic dermatitis: biomarker or simply epiphenomenon? J Dermatol (2014) 41(7):569–76. 10.1111/1346-8138.12464 24806813

[126] Hradetzky S Werfel T Rosner LM. Autoallergy in atopic dermatitis. Allergo J Int (2015) 24(1):16–22. 10.1007/s40629-015-0037-5 26120543 PMC4479480

[127] Wang HH Li YC Huang YC Efficacy of omalizumab in patients with atopic dermatitis: a systematic review and meta-analysis. J Allergy Clin Immunol (2016) 138(6):1719–22.e1. 10.1016/j.jaci.2016.05.038 27543070

[128] Holm JG Agner T Sand C Thomsen SF. Omalizumab for atopic dermatitis: case series and a systematic review of the literature. Int J Dermatol (2017) 56(1):18–26. 10.1111/ijd.13353 27337170

[129] Henault J Riggs JM Karnell JL Liarski VM Li J Shirinian L Self-reactive IgE exacerbates interferon responses associated with autoimmunity. Nat Immunol (2016) 17(2):196–203. 10.1038/ni.3326 26692173 PMC4718782

[130] Ettinger R Karnell JL Henault J Panda SK Riggs JM Kolbeck R Pathogenic mechanisms of IgE-mediated inflammation in self-destructive autoimmune responses. Autoimmunity (2017) 50(1):25–36. 10.1080/08916934.2017.1280670 28166684

[131] Pan Q Gong L Xiao H Feng Y Li L Deng Z Basophil activation-dependent autoantibody and interleukin-17 production exacerbate systemic lupus erythematosus. Front Immunol (2017) 8:348. 10.3389/fimmu.2017.00348 28396669 PMC5366357

[132] Freire PC Munoz CH Stingl G. IgE autoreactivity in bullous pemphigoid: eosinophils and mast cells as major targets of pathogenic immune reactants. Br J Dermatol (2017) 177(6):1644–53. 10.1111/bjd.15924 28868796 PMC5814899

[133] Fairley JA Baum CL Brandt DS Messingham KA Pathogenicity of IgE in autoimmunity: successful treatment of bullous pemphigoid with omalizumab. J Allergy Clin Immunol (2009) 123(3):704–5. 10.1016/j.jaci.2008.11.035 19152970 PMC4784096

[134] Dufour C Souillet AL Chaneliere C Jouen F Bodemer C Jullien D Successful management of severe infant bullous pemphigoid with omalizumab. Br J Dermatol (2012) 166(5):1140–2. 10.1111/j.1365-2133.2011.10748.x 22098309

[135] London VA Kim GH Fairley JA Woodley DT Successful treatment of bullous pemphigoid with omalizumab. Arch Dermatol (2012) 148(11):1241–3. 10.1001/archdermatol.2012.1604 23165827

[136] Yalcin AD Genc GE Celik B Gumuslu S. Anti-IgE monoclonal antibody (omalizumab) is effective in treating bullous pemphigoid and its effects on soluble CD200. Clin Lab (2014) 60(3):523–4. 10.7754/Clin.Lab.2013.130642 24697134

[137] Yu KK Crew AB Messingham KA Fairley JA Woodley DT. Omalizumab therapy for bullous pemphigoid. J Am Acad Dermatol (2014) 71(3):468–74. 10.1016/j.jaad.2014.04.053 24954907 PMC9215508

[138] Balakirski G Alkhateeb A Merk HF Leverkus M Megahed M. Successful treatment of bullous pemphigoid with omalizumab as corticosteroid-sparing agent: report of two cases and review of literature. J Eur Acad Dermatol Venereol (2016) 30(10):1778–82. 10.1111/jdv.13758 27357866

[139] Gonul MZ Keseroglu HO Ergin C Ozcan I Erdem O Bullous pemphigoid successfully treated with omalizumab. Indian J Dermatol Venereol Leprol (2016) 82(5):577–9. 10.4103/0378-6323.183628 27297272

[140] Menzinger S Kaya G Schmidt E Fontao L Laffitte E Biological and clinical response to omalizumab in a patient with bullous pemphigoid. Acta Derm Venereol (2017) 98(2):284–6. 10.2340/00015555-2845 29136264

[141] Kolkhir P Church MK Weller K Metz M Schmetzer O Maurer M Autoimmune chronic spontaneous urticaria: what we know and what we do not know. J Allergy Clin Immunol (2017) 139(6):1772–81.e1. 10.1016/j.jaci.2016.08.050 27777182

[142] Metz M Maurer M. Omalizumab in chronic urticaria. Curr Opin Allergy Clin Immunol (2012) 12(4):406–11. 10.1097/ACI.0b013e328355365a 22766620

[143] Urgert MC van den Elzen MT Knulst AC Fedorowicz Z van Zuuren EJ. Omalizumab in patients with chronic spontaneous urticaria: a systematic review and GRADE assessment. Br J Dermatol (2015) 173(2):404–15. 10.1111/bjd.13845 25891046

[144] Zhao ZT Ji CM Yu WJ Meng L Hawro T Wei JF Omalizumab for the treatment of chronic spontaneous urticaria: a meta-analysis of randomized clinical trials. J Allergy Clin Immunol (2016) 137(6):1742–50.e4. 10.1016/j.jaci.2015.12.1342 27040372

[145] Maurer M Metz M Brehler R Hillen U Jakob T Mahler V Omalizumab treatment in patients with chronic inducible urticaria: a systematic review of published evidence. J Allergy Clin Immunol (2017) 141(2):638–49. 10.1016/j.jaci.2017.06.032 28751232

[146] Maurer M Altrichter S Bieber T Biedermann T Brautigam M Seyfried S Efficacy and safety of omalizumab in patients with chronic urticaria who exhibit IgE against thyroperoxidase. J Allergy Clin Immunol (2011) 128(1):202–9.e5. 10.1016/j.jaci.2011.04.038 21636116

[147] Gericke J Metz M Ohanyan T Weller K Altrichter S Skov PS Serum autoreactivity predicts time to response to omalizumab therapy in chronic spontaneous urticaria. J Allergy Clin Immunol (2017) 139(3):1059–61.e1. 10.1016/j.jaci.2016.07.047 27838346

[148] Houser DD Arbesman CE Ito K Wicher K Cold urticaria. Immunologic studies. Am J Med (1970) 49(1):23–33. 4194010 10.1016/s0002-9343(70)80110-3

[149] Kaplan AP Garofalo J Sigler R Hauber T Idiopathic cold urticaria: in vitro demonstration of histamine release upon challenge of skin biopsies. N Engl J Med (1981) 305(18):1074–7. 10.1056/NEJM198110293051808 6168912

[150] Newcomb RW Nelson H Dermographia mediated by immunoglobulin E. Am J Med (1973) 54(2):174–80. 10.1016/0002-9343(73)90221-0 4631173

[151] Morgado-Carrasco D Fusta-Novell X Podlipnik S Combalia A Aguilera P. Clinical and photobiological response in eight patients with solar urticaria under treatment with omalizumab, and review of the literature. Photodermatol Photoimmunol Photomed (2017). 10.1111/phpp.12370 29171925

[152] Rodriguez-Jimenez P Chicharro P Perez-Plaza A de Argila D. Response to omalizumab in solar urticaria: report of 3 cases. Actas Dermosifiliogr (2017) 108(8):e53–5. 10.1016/j.ad.2016.08.011 28457471

[153] Koumaki D Seaton ED Successful treatment of refractory cholinergic urticaria with omalizumab. Int J Dermatol (2018) 57(1):114 10.1111/ijd.13808 29057464

[154] Somers EC Marder W Cagnoli P Lewis EE DeGuire P Gordon C Population-based incidence and prevalence of systemic lupus erythematosus: the michigan lupus epidemiology and surveillance program. Arthritis Rheumatol (2014) 66(2):369–78. 10.1002/art.38238 24504809 PMC4198147

[155] Dema B Charles N Pellefigues C Ricks TK Suzuki R Jiang C Immunoglobulin E plays an immunoregulatory role in lupus. J Exp Med (2014) 211(11):2159–68. 10.1084/jem.20140066 25267791 PMC4203948

[156] Yan S Chen W Wen S Zhu W Guo A Chen X Influence of component 5a receptor 1 (C5AR1) -1330T/G polymorphism on nonsedating H1-antihistamines therapy in Chinese patients with chronic spontaneous urticaria. J Dermatol Sci (2014) 76(3):240–5. 10.1016/j.jdermsci.2014.09.012 25455139

[157] Yang EM Kim SH Kim NH Park HS The genetic association of the FPRL1 promoter polymorphism with chronic urticaria in a Korean population. Ann Allergy Asthma Immunol (2010) 105(1):96–7. 10.1016/j.anai.2010.05.003 20642210

[158] Rasool R Shera IA Nissar S Yousuf Q Shah ZA IgE FcvarepsilonR1beta polymorphism and risk of developing chronic spontaneous urticaria: a study in an ethnic Kashmiri population. Allergol Immunopathol (Madr) (2015) 43(3):243–8. 10.1016/j.aller.2014.04.001 24953255

[159] Hosseini Farahabadi S Tavakkol-Afshari J Ganjali R Rafatpanah H Ghaffari J Farid-Hosseini R Association between the polymorphism of TGF-beta1 gene promoter (-509C>T) and idiopathic chronic urticaria. Iran J Allergy Asthma Immunol (2006) 5(3):109–13. 17237561

[160] Park HJ Ye YM Hur GY Kim SH Park HS. Association between a TGFbeta1 promoter polymorphism and the phenotype of aspirin-intolerant chronic urticaria in a Korean population. J Clin Pharm Ther (2008) 33(6):691–7. 10.1111/j.1365-2710.2008.00957.x 19138248

[161] Pan KY Walls RS Rajasekariah P Sherritt M Warlow RS. Polymorphism of IgE gene in chronic urticaria. Immunol Cell Biol (1996) 74(1):90–5. 10.1038/icb.1996.12 8934659

[162] Palikhe NS Kim SH Choi GS Ye YM Park HS. No evidence of association between interleukin-13 gene polymorphism in aspirin intolerant chronic urticaria. Allergy Asthma Immunol Res (2009) 1(1):36–40. 10.4168/aair.2009.1.1.36 20224668 PMC2831567

[163] Kim SH Kang YM Kim SH Cho BY Ye YM Hur GY Histamine N-methyltransferase 939A>G polymorphism affects mRNA stability in patients with acetylsalicylic acid-intolerant chronic urticaria. Allergy (2009) 64(2):213–21. 10.1111/j.1398-9995.2008.01795.x 19178400

[164] Brzoza Z Grzeszczak W Trautsolt W Moczulski D Protein tyrosine phosphatase-22 (PTPN-22) polymorphism in the pathogenesis of chronic urticaria. Allergy (2011) 66(10):1392–3. 10.1111/j.1398-9995.2011.02651.x 21599705

[165] Brzoza Z Grzeszczak W Rogala B Trautsolt W Moczulski D. PTPN22 polymorphism presumably plays a role in the genetic background of chronic spontaneous autoreactive urticaria. Dermatology (2012) 224(4):340–5. 10.1159/000339332 22722472

[166] Brzoza Z Grzeszczak W Rogala B Trautsolt W Moczulski D. CTLA-4 polymorphism in the pathogenesis of chronic spontaneous autoreactive urticaria. Allergol Immunopathol (Madr) (2014) 42(3):241–4. 10.1016/j.aller.2013.01.008 23597501

[167] Akcali C Ozkur M Erbagci Z Benlier N Aynacioglu AS. Association of insertion/deletion polymorphism of the angiotensin-converting enzyme gene with angio-oedema accompanying chronic urticaria but not chronic urticaria without angio-oedema or the autologous serum skin test response. J Eur Acad Dermatol Venereol (2008) 22(1):83–6. 10.1111/j.1468-3083.2007.02353.x 18181977

[168] Bottini N Borgiani P Otsu A Saccucci P Stefanini L Greco E IL-4 receptor alpha chain genetic polymorphism and total IgE levels in the English population: two-locus haplotypes are more informative than individual SNPs. Clin Genet (2002) 61(4):288–92. 10.1034/j.1399-0004.2002.610408.x 12030894

[169] Shin HD Park BL Kim LH Kim JS Kim JW. Interleukin-10 haplotype associated with total serum IgE in atopic dermatitis patients. Allergy (2005) 60(9):1146–51. 10.1111/j.1398-9995.2005.00839.x 16076299

[170] Takahashi N Akahoshi M Matsuda A Ebe K Inomata N Obara K Association of the IL12RB1 promoter polymorphisms with increased risk of atopic dermatitis and other allergic phenotypes. Hum Mol Genet (2005) 14(21):3149–59. 10.1093/hmg/ddi347 16159888

[171] Munthe-Kaas MC Carlsen KH Helms PJ Gerritsen J Whyte M Feijen M CTLA-4 polymorphisms in allergy and asthma and the TH1/TH2 paradigm. J Allergy Clin Immunol (2004) 114(2):280–7. 10.1016/j.jaci.2004.03.050 15316504

[172] Pykalainen M Kinos R Valkonen S Rydman P Kilpelainen M Laitinen LA Association analysis of common variants of STAT6, GATA3, and STAT4 to asthma and high serum IgE phenotypes. J Allergy Clin Immunol (2005) 115(1):80–7. 10.1016/j.jaci.2004.10.006 15637551

[173] Weidinger S Gieger C Rodriguez E Baurecht H Mempel M Klopp N Genome-wide scan on total serum IgE levels identifies FCER1A as novel susceptibility locus. PLoS Genet (2008) 4(8):e1000166. 10.1371/journal.pgen.1000166 18846228 PMC2565692

[174] Hecker M Bohnert A Konig IR Bein G Hackstein H. Novel genetic variation of human interleukin-21 receptor is associated with elevated IgE levels in females. Genes Immun (2003) 4(3):228–33. 10.1038/sj.gene.6363954 12700598

[175] Pene J Guglielmi L Gauchat JF Harrer N Woisetschlager M Boulay V IFN-gamma-mediated inhibition of human IgE synthesis by IL-21 is associated with a polymorphism in the IL-21R gene. J Immunol (2006) 177(8):5006–13. 10.4049/jimmunol.177.8.5006 17015683

[176] Ozaki K Spolski R Feng CG Qi CF Cheng J Sher A A critical role for IL-21 in regulating immunoglobulin production. Science (2002) 298(5598):1630–4. 10.1126/science.1077002 12446913

[177] Kotlarz D Zietara N Uzel G Weidemann T Braun CJ Diestelhorst J Loss-of-function mutations in the IL-21 receptor gene cause a primary immunodeficiency syndrome. J Exp Med (2013) 210(3):433–43. 10.1084/jem.20111229 23440042 PMC3600901

[178] Hysi P Kabesch M Moffatt MF Schedel M Carr D Zhang Y NOD1 variation, immunoglobulin E and asthma. Hum Mol Genet (2005) 14(7):935–41. 10.1093/hmg/ddi087 15718249

[179] Chalubinski M Grzegorczyk J Grzelak A Jarzebska M Kowalski ML The beta2-adrenoreceptor gene promoter polymorphisms may modulate beta2-agonist- and glucocorticoid-induced IgE synthesis. Allergol Immunopathol (Madr) (2014) 42(6):586–93. 10.1016/j.aller.2013.07.002 24182991

[180] Pate MB Smith JK Chi DS Krishnaswamy G. Regulation and dysregulation of immunoglobulin E: a molecular and clinical perspective. Clin Mol Allergy (2010) 8:3. 10.1186/1476-7961-8-3 20178634 PMC2837605

[181] Kabesch M Tzotcheva I Carr D Hofler C Weiland SK Fritzsch C A complete screening of the IL4 gene: novel polymorphisms and their association with asthma and IgE in childhood. J Allergy Clin Immunol (2003) 112(5):893–8. 10.1016/j.jaci.2003.08.033 14610476

[182] Maurer D Fiebiger E Reininger B Ebner C Petzelbauer P Shi GP Fc epsilon receptor I on dendritic cells delivers IgE-bound multivalent antigens into a cathepsin S-dependent pathway of MHC class II presentation. J Immunol (1998) 161(6):2731–9. 9743330

[183] Getahun A Heyman B. IgG- and IgE-mediated antigen presentation on MHC class II. Immunol Lett (2004) 92(1–2):33–8. 10.1016/j.imlet.2003.09.015 15081524

[184] Alvarez-Errico D Lessmann E Rivera J. Adapters in the organization of mast cell signaling. Immunol Rev (2009) 232(1):195–217. 10.1111/j.1600-065X.2009.00834.x 19909365 PMC3018096

[185] Campbell AM Vachier I Chanez P Vignola AM Lebel B Kochan J Expression of the high-affinity receptor for IgE on bronchial epithelial cells of asthmatics. Am J Respir Cell Mol Biol (1998) 19(1):92–7. 10.1165/ajrcmb.19.1.2648 9651184

[186] Redhu NS Saleh A Lee HC Halayko AJ Ziegler SF Gounni AS IgE induces transcriptional regulation of thymic stromal lymphopoietin in human airway smooth muscle cells. J Allergy Clin Immunol (2011) 128(4):892–6.e2. 10.1016/j.jaci.2011.06.045 21835441

[187] Shibaki A. Fc epsilon RI on dendritic cells: a receptor, which links IgE mediated allergic reaction and T cell mediated cellular response. J Dermatol Sci (1998) 20(1):29–38. 10.1016/S0923-1811(99)00003-1 10342746

[188] Karagiannis SN Bracher MG Beavil RL Beavil AJ Hunt J McCloskey N Role of IgE receptors in IgE antibody-dependent cytotoxicity and phagocytosis of ovarian tumor cells by human monocytic cells. Cancer Immunol Immunother (2008) 57(2):247–63. 10.1007/s00262-007-0371-7 17657488 PMC11030264

[189] Shin JS Greer AM The role of FcepsilonRI expressed in dendritic cells and monocytes. Cell Mol Life Sci (2015) 72(12):2349–60. 10.1007/s00018-015-1870-x 25715742 PMC4479177

[190] Gounni AS Lamkhioued B Ochiai K Tanaka Y Delaporte E Capron A High-affinity IgE receptor on eosinophils is involved in defence against parasites. Nature (1994) 367(6459):183–6. 10.1038/367183a0 8114916

[191] Alphonse MP Saffar AS Shan L HayGlass KT Simons FE Gounni AS. Regulation of the high affinity IgE receptor (Fc epsilonRI) in human neutrophils: role of seasonal allergen exposure and Th-2 cytokines. PLoS One (2008) 3(4):e1921. 10.1371/journal.pone.0001921 18382690 PMC2275309

[192] Joseph M Gounni AS Kusnierz JP Vorng H Sarfati M Kinet JP Expression and functions of the high-affinity IgE receptor on human platelets and megakaryocyte precursors. Eur J Immunol (1997) 27(9):2212–8. 10.1002/eji.1830270914 9341761

[193] Hasegawa S Pawankar R Suzuki K Nakahata T Furukawa S Okumura K Functional expression of the high affinity receptor for IgE (FcepsilonRI) in human platelets and its’ intracellular expression in human megakaryocytes. Blood (1999) 93(8):2543–51. 10194433

[194] Charles N Hardwick D Daugas E Illei GG Rivera J. Basophils and the T helper 2 environment can promote the development of lupus nephritis. Nat Med (2010) 16(6):701–7. 10.1038/nm.2159 20512127 PMC2909583

[195] Reich K Heine A Hugo S Blaschke V Middel P Kaser A Engagement of the Fc epsilon RI stimulates the production of IL-16 in langerhans cell-like dendritic cells. J Immunol (2001) 167(11):6321–9. 10.4049/jimmunol.167.11.6321 11714796

[196] Galli SJ Tsai M. IgE and mast cells in allergic disease. Nat Med (2012) 18(5):693–704. 10.1038/nm.2755 22561833 PMC3597223

[197] MacGlashan DW Jr Bochner BS Adelman DC Jardieu PM Togias A McKenzie-White J Down-regulation of Fc(epsilon)RI expression on human basophils during in vivo treatment of atopic patients with anti-IgE antibody. J Immunol (1997) 158(3):1438–45. 9013989

[198] Prussin C Griffith DT Boesel KM Lin H Foster B Casale TB. Omalizumab treatment downregulates dendritic cell FcepsilonRI expression. J Allergy Clin Immunol (2003) 112(6):1147–54. 10.1016/j.jaci.2003.10.003 14657874

[199] Metz M Staubach P Bauer A Brehler R Gericke J Kangas M Clinical efficacy of omalizumab in chronic spontaneous urticaria is associated with a reduction of FcεRI-positive cells in the skin. Theranostics (2017) 7(5):1266–76. 10.7150/thno.18304 28435464 PMC5399592

[200] Kitaura J Kinoshita T Matsumoto M Chung S Kawakami Y Leitges M IgE- and IgE+Ag-mediated mast cell migration in an autocrine/paracrine fashion. Blood (2005) 105(8):3222–9. 10.1182/blood-2004-11-4205 15637135 PMC1464406

[201] Kitaura J Song J Tsai M Asai K Maeda-Yamamoto M Mocsai A Evidence that IgE molecules mediate a spectrum of effects on mast cell survival and activation via aggregation of the FcepsilonRI. Proc Natl Acad Sci U S A (2003) 100(22):12911–6. 10.1073/pnas.1735525100 14569021 PMC240718

[202] Kashiwakura J Okayama Y Furue M Kabashima K Shimada S Ra C Most highly cytokinergic IgEs have polyreactivity to autoantigens. Allergy Asthma Immunol Res (2012) 4(6):332–40. 10.4168/aair.2012.4.6.332 23115729 PMC3479226

[203] Bax HJ Bowen H Dodev TS Sutton BJ Gould HJ. Mechanism of the antigen-independent cytokinergic SPE-7 IgE activation of human mast cells in vitro. Sci Rep (2015) 5:9538. 10.1038/srep09538 25892150 PMC4402612

[204] Greer AM Wu N Putnam AL Woodruff PG Wolters P Kinet JP Serum IgE clearance is facilitated by human FcepsilonRI internalization. J Clin Invest (2014) 124(3):1187–98. 10.1172/JCI68964 24569373 PMC3938266

[205] Perrigoue JG Saenz SA Siracusa MC Allenspach EJ Taylor BC Giacomin PR MHC class II-dependent basophil-CD4+ T cell interactions promote T(H)2 cytokine-dependent immunity. Nat Immunol (2009) 10(7):697–705. 10.1038/ni.1740 19465906 PMC2711559

[206] Yoshimoto T Yasuda K Tanaka H Nakahira M Imai Y Fujimori Y Basophils contribute to T(H)2-IgE responses in vivo via IL-4 production and presentation of peptide-MHC class II complexes to CD4+ T cells. Nat Immunol (2009) 10(7):706–12. 10.1038/ni.1737 19465908

[207] Platzer B Stout M Fiebiger E. Functions of dendritic-cell-bound IgE in allergy. Mol Immunol (2015) 68(2 Pt A):116–9. 10.1016/j.molimm.2015.05.016 26052071 PMC4623942

[208] Bayry J. Lupus pathogenesis: role of IgE autoantibodies. Cell Res (2016) 26(3):271–2. 10.1038/cr.2016.12 26794873 PMC4783465

[209] Le T Tversky J Chichester KL Bieneman AP Huang SK Wood RA Interferons modulate Fc epsilon RI-dependent production of autoregulatory IL-10 by circulating human monocytoid dendritic cells. J Allergy Clin Immunol (2009) 123(1):217–23. 10.1016/j.jaci.2008.09.013 18845324 PMC2674243

[210] Larson D Hubner MP Torrero MN Morris CP Brankin A Swierczewski BE Chronic helminth infection reduces basophil responsiveness in an IL-10-dependent manner. J Immunol (2012) 188(9):4188–99. 10.4049/jimmunol.1101859 22461700 PMC3331970

[211] Pyle DM Yang VS Gruchalla RS Farrar JD Gill MA. IgE cross-linking critically impairs human monocyte function by blocking phagocytosis. J Allergy Clin Immunol (2013) 131(2):491–500.e1–5. 10.1016/j.jaci.2012.11.037 23374271 PMC3564054

[212] Mellor AL Munn DH. IDO expression by dendritic cells: tolerance and tryptophan catabolism. Nat Rev Immunol (2004) 4(10):762–74. 10.1038/nri1457 15459668

[213] Dehlink E Platzer B Baker AH Larosa J Pardo M Dwyer P A soluble form of the high affinity IgE receptor, Fc-epsilon-RI, circulates in human serum. PLoS One (2011) 6(4):e19098. 10.1371/journal.pone.0019098 21544204 PMC3081330

[214] Platzer B Ruiter F van der Mee J Fiebiger E Soluble IgE receptors – elements of the IgE network. Immunol Lett (2011) 141(1):36–44. 10.1016/j.imlet.2011.08.004 21920387 PMC3205341

[215] Gould HJ Sutton BJ. IgE in allergy and asthma today. Nat Rev Immunol (2008) 8(3):205–17. 10.1038/nri2273 18301424

[216] Vercelli D Helm B Marsh P Padlan E Geha RS Gould H. The B-cell binding site on human immunoglobulin E. Nature (1989) 338(6217):649–51. 10.1038/338649a0 2468089

[217] Weskamp G Ford JW Sturgill J Martin S Docherty AJ Swendeman S ADAM10 is a principal ‘sheddase’ of the low-affinity immunoglobulin E receptor CD23. Nat Immunol (2006) 7(12):1293–8. 10.1038/ni1399 17072319

[218] Bansal A Roberts T Hay EM Kay R Pumphrey RS Wilson PB Soluble CD23 levels are elevated in the serum of patients with primary Sjogren’s syndrome and systemic lupus erythematosus. Clin Exp Immunol (1992) 89(3):452–5. 10.1111/j.1365-2249.1992.tb06979.x 1387597 PMC1554466

[219] Acharya M Borland G Edkins AL Maclellan LM Matheson J Ozanne BW CD23/FcepsilonRII: molecular multi-tasking. Clin Exp Immunol (2010) 162(1):12–23. 10.1111/j.1365-2249.2010.04210.x 20831712 PMC2990925

[220] Mudde GC Bheekha R Bruijnzeel-Koomen CA. Consequences of IgE/CD23-mediated antigen presentation in allergy. Immunol Today (1995) 16(8):380–3. 10.1016/0167-5699(95)80005-0 7546193

[221] Cornaby C Gibbons L Mayhew V Sloan CS Welling A Poole BD. B cell epitope spreading: mechanisms and contribution to autoimmune diseases. Immunol Lett (2015) 163(1):56–68. 10.1016/j.imlet.2014.11.001 25445494

[222] Jensen-Jarolim E Singer J Why could passive Immunoglobulin E antibody therapy be safe in clinical oncology? Clin Exp Allergy (2011) 41(10):1337–40. 10.1111/j.1365-2222.2011.03764.x 21545551 PMC4429181

[223] Liu FT Rabinovich GA. Galectins: regulators of acute and chronic inflammation. Ann N Y Acad Sci (2010) 1183:158–82. 10.1111/j.1749-6632.2009.05131.x 20146714

[224] Robertson MW Albrandt K Keller D Liu FT. Human IgE-binding protein: a soluble lectin exhibiting a highly conserved interspecies sequence and differential recognition of IgE glycoforms. Biochemistry (1990) 29(35):8093–100. 10.1021/bi00487a015 2261464

[225] de Oliveira FL Gatto M Bassi N Luisetto R Ghirardello A Punzi L Galectin-3 in autoimmunity and autoimmune diseases. Exp Biol Med (Maywood) (2015) 240(8):1019–28. 10.1177/1535370215593826 26142116 PMC4935288

[226] Sanz ML Prieto I Garcia BE Oehling A. Diagnostic reliability considerations of specific IgE determination. J Investig Allergol Clin Immunol (1996) 6(3):152–61. 8807505

[227] Drinkwater N Cossins BP Keeble AH Wright M Cain K Hailu H Human immunoglobulin E flexes between acutely bent and extended conformations. Nat Struct Mol Biol (2014) 21(4):397–404. 10.1038/nsmb.2795 24632569 PMC3977038

[228] Maurer D Fiebiger E Reininger B Wolff-Winiski B Jouvin MH Kilgus O Expression of functional high affinity immunoglobulin E receptors (Fc epsilon RI) on monocytes of atopic individuals. J Exp Med (1994) 179(2):745–50. 10.1084/jem.179.2.745 8294882 PMC2191351

[229] Yamaguchi M Sayama K Yano K Lantz CS Noben-Trauth N Ra C IgE enhances Fc epsilon receptor I expression and IgE-dependent release of histamine and lipid mediators from human umbilical cord blood-derived mast cells: synergistic effect of IL-4 and IgE on human mast cell Fc epsilon receptor I expression and mediator release. J Immunol (1999) 162(9):5455–65. 10228025

[230] Oda M Kozono H Morii H Azuma T. Evidence of allosteric conformational changes in the antibody constant region upon antigen binding. Int Immunol (2003) 15(3):417–26. 10.1093/intimm/dxg036 12618486

[231] Kochuyt AM. Sensitivity and specificity of food specific IgE and IgG determinations for the diagnosis of food allergy. Acta Gastroenterol Belg (2006) 69(1):43–8. 16673562

[232] Zimina EP Fritsch A Schermer B Bakulina AY Bashkurov M Benzing T Extracellular phosphorylation of collagen XVII by ecto-casein kinase 2 inhibits ectodomain shedding. J Biol Chem (2007) 282(31):22737–46. 10.1074/jbc.M701937200 17545155

[233] Panneerselvam J Shanker M Jin J Branch CD Muralidharan R Zhao YD Phosphorylation of interleukin (IL)-24 is required for mediating its anti-cancer activity. Oncotarget (2015) 6(18):16271–86. 10.18632/oncotarget.3977 26009991 PMC4599269

[234] Zimina EP Hofmann SC Fritsch A Kern JS Sitaru C Bruckner-Tuderman L Bullous pemphigoid autoantibodies preferentially recognize phosphoepitopes in collagen XVII. J Invest Dermatol (2008) 128(11):2736–9. 10.1038/jid.2008.132 18548115

[235] Schreiber A Rolle S Peripelittchenko L Rademann J Schneider W Luft FC Phosphoinositol 3-kinase-gamma mediates antineutrophil cytoplasmic autoantibody-induced glomerulonephritis. Kidney Int (2010) 77(2):118–28. 10.1038/ki.2009.420 19907415

[236] Sircar G Jana K Dasgupta A Saha S Gupta Bhattacharya S. Epitope mapping of Rhi o 1 and generation of a hypoallergenic variant: a candidate molecule for fungal allergy vaccines. J Biol Chem (2016) 291(34):18016–29. 10.1074/jbc.M116.732032 27358405 PMC5016188

[237] Yeh CC Tai HY Chou H Wu KG Shen HD. Vacuolar serine protease is a major allergen of *Fusarium proliferatum* and an IgE-cross reactive pan-fungal allergen. Allergy Asthma Immunol Res (2016) 8(5):438–44. 10.4168/aair.2016.8.5.438 27334782 PMC4921698

[238] Donovan GR Baldo BA. Crossreactivity of IgE antibodies from sera of subjects allergic to both ryegrass pollen and wheat endosperm proteins: evidence for common allergenic determinants. Clin Exp Allergy (1990) 20(5):501–9. 10.1111/j.1365-2222.1990.tb03142.x 2253081

[239] Jappe U Raulf-Heimsoth M Hoffmann M Burow G Hubsch-Muller C Enk A. In vitro hymenoptera venom allergy diagnosis: improved by screening for cross-reactive carbohydrate determinants and reciprocal inhibition. Allergy (2006) 61(10):1220–9. 10.1111/j.1398-9995.2006.01232.x 16942573

[240] Mahler V Gutgesell C Valenta R Fuchs T. Natural rubber latex and hymenoptera venoms share ImmunoglobinE-epitopes accounting for cross-reactive carbohydrate determinants. Clin Exp Allergy (2006) 36(11):1446–56. 10.1111/j.1365-2222.2006.02587.x 17083355

[241] Carballada FJ Gonzalez-Quintela A Nunez-Orjales R Vizcaino L Boquete M. Double (honeybee and wasp) immunoglobulin E reactivity in patients allergic to hymenoptera venom: the role of cross-reactive carbohydrates and alcohol consumption. J Investig Allergol Clin Immunol (2010) 20(6):484–9. 21243932

[242] Pham NH Baldo BA. Allergenic relationship between taxonomically diverse pollens. Clin Exp Allergy (1995) 25(7):599–606. 10.1111/j.1365-2222.1995.tb01107.x 8521178

[243] Twardosz A Hayek B Seiberler S Vangelista L Elfman L Gronlund H Molecular characterization, expression in *Escherichia coli*, and epitope analysis of a two EF-hand calcium-binding birch pollen allergen, Bet v 4. Biochem Biophys Res Commun (1997) 239(1):197–204. 10.1006/bbrc.1997.6860 9345295

[244] Oberhuber C Ma Y Wopfner N Gadermaier G Dedic A Niggemann B Prevalence of IgE-binding to Art v 1, Art v 4 and Amb a 1 in mugwort-allergic patients. Int Arch Allergy Immunol (2008) 145(2):94–101. 10.1159/000108134 17823540

[245] Tinghino R Twardosz A Barletta B Puggioni EM Iacovacci P Butteroni C Molecular, structural, and immunologic relationships between different families of recombinant calcium-binding pollen allergens. J Allergy Clin Immunol (2002) 109(2):314–20. 10.1067/mai.2002.121528 11842303

[246] Mares-Mejia I Martinez-Caballero S Garay-Canales C Cano-Sanchez P Torres-Larios A Lara-Gonzalez S Structural insights into the IgE mediated responses induced by the allergens Hev b 8 and Zea m 12 in their dimeric forms. Sci Rep (2016) 6:32552. 10.1038/srep32552 27586352 PMC5009318

[247] Chelminska M Specjalski K Rozylo A Kolakowska A Jassem E. Differentiating of cross-reactions in patients with latex allergy with the use of ISAC test. Postepy Dermatol Alergol (2016) 33(2):120–7. 10.5114/ada.2016.59154 27279821 PMC4884780

[248] Aalberse RC Crameri R. IgE-binding epitopes: a reappraisal. Allergy (2011) 66(10):1261–74. 10.1111/j.1398-9995.2011.02656.x 21623828

[249] Fahlbusch B Rudeschko O Schumann C Steurich F Henzgen M Schlenvoigt G Further characterization of IgE-binding antigens in kiwi, with particular emphasis on glycoprotein allergens. J Investig Allergol Clin Immunol (1998) 8(6):325–32. 10028478

[250] Son DY Scheurer S Hoffmann A Haustein D Vieths S. Pollen-related food allergy: cloning and immunological analysis of isoforms and mutants of Mal d 1, the major apple allergen, and Bet v 1, the major birch pollen allergen. Eur J Nutr (1999) 38(4):201–15. 10.1007/s003940050063 10502033

[251] Gavrovic-Jankulovic M Cirkovic T Burazer L Vuckovic O Jankov RM. IgE cross-reactivity between meadow fescue pollen and kiwi fruit in patients’ sera with sensitivity to both extracts. J Investig Allergol Clin Immunol (2002) 12(4):279–86. 12926188

[252] Cudowska B Kaczmarski M Restani P. Immunoblotting in the diagnosis of cross-reactivity in children allergic to birch. Rocz Akad Med Bialymst (2005) 50:268–73. 16358981

[253] Iraneta SG Seoane MA Laucella SA Apicella C Alonso A Duschak VG. Antigenicity and immunocrossreactivity of orange tree pollen and orange fruit allergenic extracts. Int Arch Allergy Immunol (2005) 137(4):265–72. 10.1159/000086419 15970633

[254] Madhurantakam C Nilsson OB Uchtenhagen H Konradsen J Saarne T Hogbom E Crystal structure of the dog lipocalin allergen Can f 2: implications for cross-reactivity to the cat allergen Fel d 4. J Mol Biol (2010) 401(1):68–83. 10.1016/j.jmb.2010.05.043 20621650

[255] Apostolovic D Sanchez-Vidaurre S Waden K Curin M Grundstrom J Gafvelin G The cat lipocalin Fel d 7 and its cross-reactivity with the dog lipocalin Can f 1. Allergy (2016) 71(10):1490–5. 10.1111/all.12955 27289080

[256] Hemmer W Klug C Swoboda I. Update on the bird-egg syndrome and genuine poultry meat allergy. Allergo J Int (2016) 25:68–75. 10.1007/s40629-016-0108-2 27340614 PMC4861744

[257] Kuehn A Codreanu-Morel F Lehners-Weber C Doyen V Gomez-Andre SA Bienvenu F Cross-reactivity to fish and chicken meat – a new clinical syndrome. Allergy (2016) 71(12):1772–81. 10.1111/all.12968 27344988

[258] Jeong KY Son M Park JH Park KH Park HJ Lee JH Cross-reactivity between oak and birch pollens in Korean tree pollinosis. J Korean Med Sci (2016) 31(8):1202–7. 10.3346/jkms.2016.31.8.1202 27478329 PMC4951548

[259] Doenhoff MJ El-Faham M Liddell S Fuller HR Stanley RG Schramm G Cross-reactivity between *Schistosoma mansoni* antigens and the latex allergen Hev b 7: putative implication of cross-reactive carbohydrate determinants (CCDs). PLoS One (2016) 11(7):e0159542. 10.1371/journal.pone.0159542 27467385 PMC4965158

[260] Santiago Hda C Nutman TB. Role in allergic diseases of immunological cross-reactivity between allergens and homologues of parasite proteins. Crit Rev Immunol (2016) 36(1):1–11. 10.1615/CritRevImmunol.2016016545 27480900 PMC5438203

[261] Utsch L Logiantara A Wallner M Hofer H van Ree R van Rijt LS. Birch pollen immunotherapy inhibits anaphylaxis to the cross-reactive apple allergen Mal d 1 in mice. Clin Exp Allergy (2016) 46(11):1474–83. 10.1111/cea.12775 27376790

[262] Geroldinger-Simic M Kinaciyan T Nagl B Baumgartner-Durchschlag U Huber H Ebner C Oral exposure to Mal d 1 affects the immune response in patients with birch pollen allergy. J Allergy Clin Immunol (2013) 131(1):94–102. 10.1016/j.jaci.2012.06.039 22921871

[263] Matricardi PM Kleine-Tebbe J Hoffmann HJ Valenta R Hilger C Hofmaier S EAACI molecular allergology user’s guide. Pediatr Allergy Immunol (2016) 27(Suppl 23):1–250. 10.1111/pai.12563 27288833

